# Manifold-aware synthesis of high-resolution diffusion from structural imaging

**DOI:** 10.3389/fnimg.2022.930496

**Published:** 2022-09-08

**Authors:** Benoit Anctil-Robitaille, Antoine Théberge, Pierre-Marc Jodoin, Maxime Descoteaux, Christian Desrosiers, Hervé Lombaert

**Affiliations:** ^1^The Shape Lab, Department of Computer and Software Engineering, ETS Montreal, Montreal, QC, Canada; ^2^Sherbrooke Connectivity Imaging Laboratory (SCIL), Department of Computer Science, Sherbrooke University, Sherbrooke, QC, Canada

**Keywords:** diffusion synthesis, manifold-valued data learning, 3D IRM, brain imaging, Riemannian geometry

## Abstract

The physical and clinical constraints surrounding diffusion-weighted imaging (DWI) often limit the spatial resolution of the produced images to voxels up to eight times larger than those of T1w images. The detailed information contained in accessible high-resolution T1w images could help in the synthesis of diffusion images with a greater level of detail. However, the non-Euclidean nature of diffusion imaging hinders current deep generative models from synthesizing physically plausible images. In this work, we propose the first Riemannian network architecture for the direct generation of diffusion tensors (DT) and diffusion orientation distribution functions (dODFs) from high-resolution T1w images. Our integration of the log-Euclidean Metric into a learning objective guarantees, unlike standard Euclidean networks, the mathematically-valid synthesis of diffusion. Furthermore, our approach improves the fractional anisotropy mean squared error (FA MSE) between the synthesized diffusion and the ground-truth by more than 23% and the cosine similarity between principal directions by almost 5% when compared to our baselines. We validate our generated diffusion by comparing the resulting tractograms to our expected real data. We observe similar fiber bundles with streamlines having <3% difference in length, <1% difference in volume, and a visually close shape. While our method is able to generate diffusion images from structural inputs in a high-resolution space within 15 s, we acknowledge and discuss the limits of diffusion inference solely relying on T1w images. Our results nonetheless suggest a relationship between the high-level geometry of the brain and its overall white matter architecture that remains to be explored.

## 1. Introduction

Diffusion MRI is of crucial importance in multiple challenging tasks, including the diagnosis of complex cognitive disorders (Neuner et al., 2010; Kantarci et al., 2017; Kelly et al., 2018), the study of neurodegenerative diseases (Huang et al., 2007; Gattellaro et al., 2009) and neurosurgical planning (Costabile et al., 2019). Nonetheless, diffusion-weighted imaging (DWI) suffers from a low signal-to-noise ratio (SNR) and a poor spatial resolution arising from physical and clinical limitations such as the use of echo-planar imaging (EPI) and limited patient scanning time. Indeed, the induced trade-off between image resolution, SNR and imaging time in the acquisition of DW often results in images with voxel size up to 8 times larger than other common modalities such as structural T1w images, e.g., 1 mm iso. for DWI vs. 2 mm iso. for T1w (Poot et al., 2013). Hence, it has been shown that the increased voxel size in DW images can impair their subsequent analysis (Alexander et al., 2001; Oouchi et al., 2007).

These limitations have triggered the development of post-processing methods that aim to improve the spatial resolution of low-resolution diffusion volumes. Tackling the estimation of high-resolution (HR) diffusion from low-resolution (LR) images was first explored with interpolation-based methods (Arsigny et al., 2006; Dyrby et al., 2014; Yap et al., 2014). These approaches resample existing images to a higher-resolution grid and is nowadays a default step in diffusion MRI processing tools such as in TractoFlow (Theaud et al., 2020) and MRtrix3 (Tournier et al., 2019). Although fine anatomical details can be enhanced with such technique, interpolations will always be limited by the inherent coarseness of the original diffusion data as they exclusively rely on intra-image information.

Machine learning offers an effective way to leverage the rich information contained in HR images for the synthesis of diffusion imaging in the same resolution, thus going beyond interpolation. In Alexander et al. (2017), a fully supervised image quality transfer (IQT) framework using random forests is proposed to learn a non-linear mapping between paired low-quality and high-quality diffusion data. Similarly, in Elsaid and Wu (2019), a supervised 2D SRCNN is used for the same objective. The authors demonstrate that learning a mapping from an LR input to its HR version not only helps recovering anatomical details better than interpolation, but can also help in downstream tasks such as tractography. However, such approaches rely on limited high-resolution diffusion data which are costly and challenging to acquire. Moreover, the methods in Alexander et al. (2017) and Elsaid and Wu (2019) have only been tested on small datasets comprising a maximum of 23 subjects and three subjects, respectively.

In parallel, deep neural networks offer unsupervised learning techniques that only require few paired training samples to train specific synthesis tasks. More particularly, Generative Adversarial Networks (GANs) (Goodfellow et al., 2014) have been successfully used for the synthesis of missing modalities (Dar et al., 2019), image-to-image translation (Zhu et al., 2017; Lei et al., 2019), and image super-resolution (Sánchez and Vilaplana, 2018), just to name a few. Therefore, deep generative models could be a key solution for the synthesis of high-resolution diffusion from unpaired images expressing a higher level of structural details such as T1w images, thus removing the dependency of the algorithms to costly high-resolution diffusion images.

The synthesis of raw DWI signals with a proper angular resolution is a resource-intensive task. Indeed, high-angular resolution diffusion volumes typically have a few dozen to a few hundreds channels, each representing an acquisition in a particular orientation. To alleviate this potential computational burden, the direct generation of more compact diffusion models, notably Diffusion Tensor (DT) or Orientation Distribution Functions (ODFs) is an interesting avenue. However, current deep learning architectures struggle to generate such reconstruction schemes because of their non-Euclidean nature (Huang et al., 2019). Indeed, each voxel of a DT image lies on a Riemannian manifold of symmetric positive definite (SPD) 3×3 matrices (Arsigny et al., 2006), and ODFs can be represented as points on an n-Sphere manifold 𝕊^*n*^ (Cheng et al., 2009). In the context of image synthesis, the inability of networks to capture the underlying non-linear Riemannian manifold geometry of the data results in the generation of implausible images that miss the important mathematical properties of diffusion imaging (Huang et al., 2019). Consequently, the limitations of current deep neural networks (DNN) have impeded the development of generative models in diffusion imaging, which have been mostly restricted to the synthesis of DT scalar maps such as Fractional Anisotropy (FA) and Mean Diffusivity (MD).

### 1.1. Structural-to-diffusion synthesis

Amidst the literature, Gu et al. (2019) study the generation of diffusion-derived scalar maps from downsampled structural images. To do so, the authors use a CycleGAN to learn the intermodal relationships between T1w images and FA/MD maps and successfully translate one to another. They demonstrate that structural images share sufficient information with the diffusion anisotropy of tissues to synthesize plausible 2D FA and MD slices. Similarly, in Lan et al. (2021), a Self-attention Conditional GAN (SC-GAN) is used to generate FA and MD maps from different input modalities including structural T1w images. Their results indicate that both the 3D contextual information and the adversarial objective are important building blocks for the synthesis of diffusion data. In Zhong et al. (2020), dual GANs with a Markovian discriminator (Li and Wand, 2016) are employed for the harmonization of inter-site DT-derived metrics. Finally, in Son et al. (2019) functional MRI in combination with structural T1w inputs are fed to a CNN network to generate DT. This body of work demonstrates the potential of generative models for the structural-to-diffusion synthesis of imaging data. Nevertheless, they remain limited (Gu et al., 2019; Son et al., 2019; Lan et al., 2021) in not exploiting the high-resolution information contained in the structural images to their full extent by either considering downsampled version of the T1w inputs or 2D slices with limited context. In addition, even though diffusion scalar maps are clinically useful, they mostly ignore fiber orientations and are of limited interest for tasks such as tractography or connectome visualization. With regards to the generated tensors in Son et al. (2019), the authors provide no guarantee on their mathematical validity, such as symmetric positive definiteness, nor on their usability in a downstream task such as tractography.

### 1.2. Manifold-valued data learning

Deep learning models are well suited to model data lying in an Euclidean vector space. However, the Euclidean operations from which they are built upon, e.g., convolutions or pooling, are not well defined on curved manifolds. Moreover, the application of Euclidean geometry to manifold-valued data, such as DT and ODF, has well-documented side effects (Arsigny et al., 2006). Consequently, studies that use neural networks for the accurate processing of data on Riemannian manifolds have started to emerge (Brooks et al., 2019; Chakraborty et al., 2019). However, these works require substantial modifications of known deep learning models and call for further investigation in a broader set of scenarios.

Another avenue for the processing of manifold-valued data resides in the design of computationally efficient Riemannian metrics. To that purpose, Arsigny et al. (2006) proposed a log-Euclidean metric to process data lying on the $S_{++}^{*}$ manifold with applications to diffusion tensors. With the help of the log and exp maps defined in Arsigny et al. (2006), one can process tensors using Euclidean operations and guarantee that the processed tensors keep their SPD properties. Likewise, a log-Euclidean framework has also been proposed in Cheng et al. (2009) for the computation of orientation distribution function and applied to diffusion ODF. These two frameworks, combined with the matrix backpropagation of spectral layers presented in Ionescu et al. (2015), constitutes the fundamentals of the following manifold-valued data learning approaches. For instance, in Huang and Van Gool (2017), the authors have integrated the log-Euclidean metric into their deep learning model called SPDNet to learn compact and discriminative SPD matrices. Although SPDNet offers a way to learn data on $S_{++}^{*}$, it has not been designed for spatially organized and volumetric SPD matrices learning as in DT.

More recently, Huang et al. (2019) proposed a Wasserstein GAN (WGAN) (Arjovsky et al., 2017) leveraging the log-Euclidean metric to synthesize plausible DT, among other manifold-valued data type. To ensure the validity of the generated data, the authors project the output of their generator network to an Euclidean space using the log(·) map in Arsigny et al. (2006) prior to the discriminator assessment. The exp(·) operation is then used on the synthesized output to recover valid DT. Despite its ability to generate mathematically valid diffusion, the model in Huang et al. (2019) outputs images that are not conditioned by any real subject specific information (e.g., a T1w image) and, thus, are less clinically valuable. In addition, this prior work only focuses on the generation of DT in 2D, which once again limits the value of the generated data.

### 1.3. Contributions

**Figure 1:**
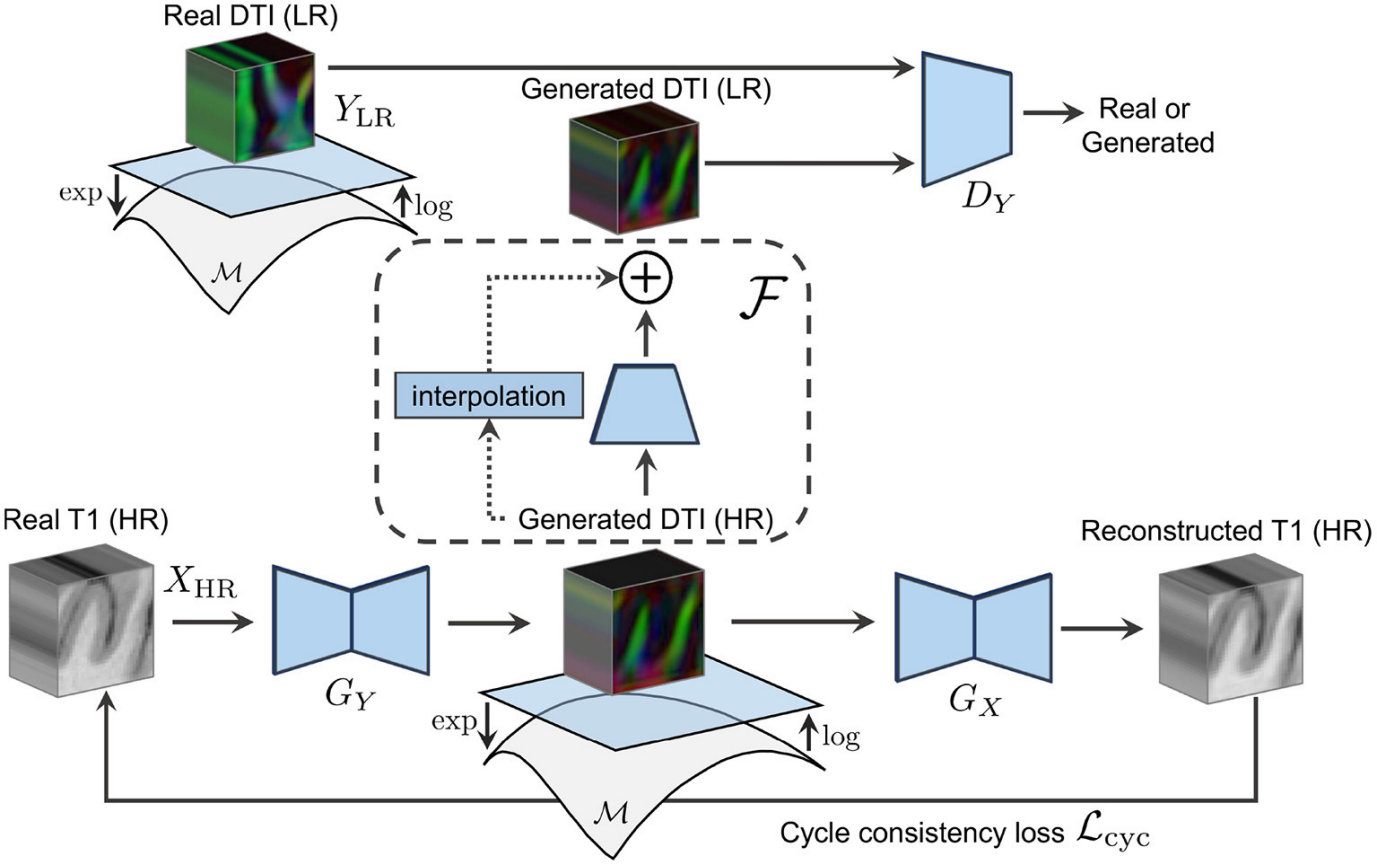
The forward cycle of our manifold-aware CycleGAN. *G*_*Y*_ generates high-resolution DT/ODF in the log-Euclidean domain using the Riemannian maps log(·) and exp(·). *D*_*Y*_ assesses the generated images quality and provides feedback to *G*_*Y*_. *G*_*X*_ tries to reconstruct the original T1w images from log(exp(*G*_*Y*_(**x**))).

This work proposes a novel deep learning architecture that leverages the detailed information of high-resolution structural images to guide the synthesis of DT and ODF in the same high-resolution space as shown in Figure 1. Based on the CycleGAN architecture (Zhu et al., 2017), our solution exploits the inherent cross-modality representations of structural and diffusion images to learn functions that map one to another in a weakly supervised manner. To do so, we address the current limitations of deep learning models built upon Euclidean operations by integrating a Riemannian framework, namely the log-Euclidean framework, for statistical computations on DT and ODF directly into the model. Such Riemannian framework within the learning procedure of the network enforces a valid synthesis of diffusion data that lies on a desired Riemannian manifold. By constraining the generated diffusion to lie on the Riemannian manifold of 3 × 3 symmetric positive definite matrices ($S_{++}^{3}$) for DT and on the n-Sphere 𝕊^**n**^ manifold for ODF, we guarantee the mathematical coherence of the solution. As opposed to existing deep generative models that focused on the generation of diffusion derived scalar maps (Li and Wand, 2016; Gu et al., 2019; Lan et al., 2021), our model outputs complete diffusion schemes, here the 3 × 3 DT and the spherical harmonic coefficients of ODF. This important difference allows one to generate, within ranges of accuracy, tractography, tractogram visualization, and fiber bundle segmentation in addition to scalar maps computation directly from our network output when only a T1w image is available as input.

Specifically, our contributions are as follows:

- The first Riemannian network for the cycle-consistent mapping between real-valued images and data lying on both the $S_{++}^{3}$ and the 𝕊^**n**^ manifolds;
- The first deep learning model for the guided super-resolution of DT and ODF from unpaired high-resolution structural images and limited priors;
- A comprehensive analysis of synthesized diffusion from structural imaging including the evaluation of full-valued diffusion data, scalar maps and tractography.

## 2. Materials and methods

**Figure 2:**
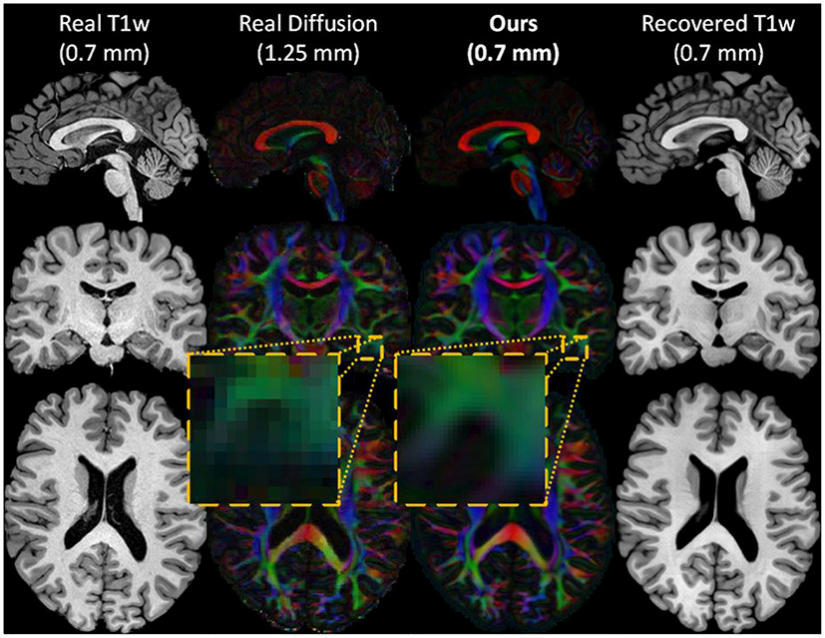
An example of the inputs and outputs of the forward cycle of our network. The HR T1w input image **(first column)**, the real LR diffusion **(second column)**, the generated HR diffusion **(third column)**, and the recovered HR T1w image **(last column)** of a test subject. During the forward cycle, an HR T1w image is translated to an HR diffusion image at the same spatial resolution. The original HR T1w image is then restored from the synthesized HR diffusion image.

Let *X* be the real-valued domain of structural images (e.g., T1w) and *Y* be the manifold-valued domain of diffusion images (e.g., DT or ODF). We aim at learning mapping functions *G*_*Y*_ : *X*_HR_ ↦ *Y*_HR_ and *G*_*X*_ : *Y*_HR_ ↦ *X*_HR_ that translate high-resolution T1w images to high-resolution diffusion images (see Figure 2) and the other way around. Learning such mapping functions is typically done using training pairs (**x**, **y**) which, in our case, correspond to HR T1w and HR diffusion data of the *same* subject aligned to a common space. In practice, obtaining this paired data is challenging as it requires subjects to undergo multiple MRI scans. As a result, we mainly have access to *unpaired* HR T1w images and LR diffusion data that, unlike for paired examples, come from *different* subjects and are acquired at different spatial resolutions. To address this problem, we propose a Manifold-Aware CycleGAN (MA-CycleGAN) architecture that inherently handles both the domain translation and the super-resolution of diffusion, while accounting for the Riemannian geometry of the data.

**Figure 3:**
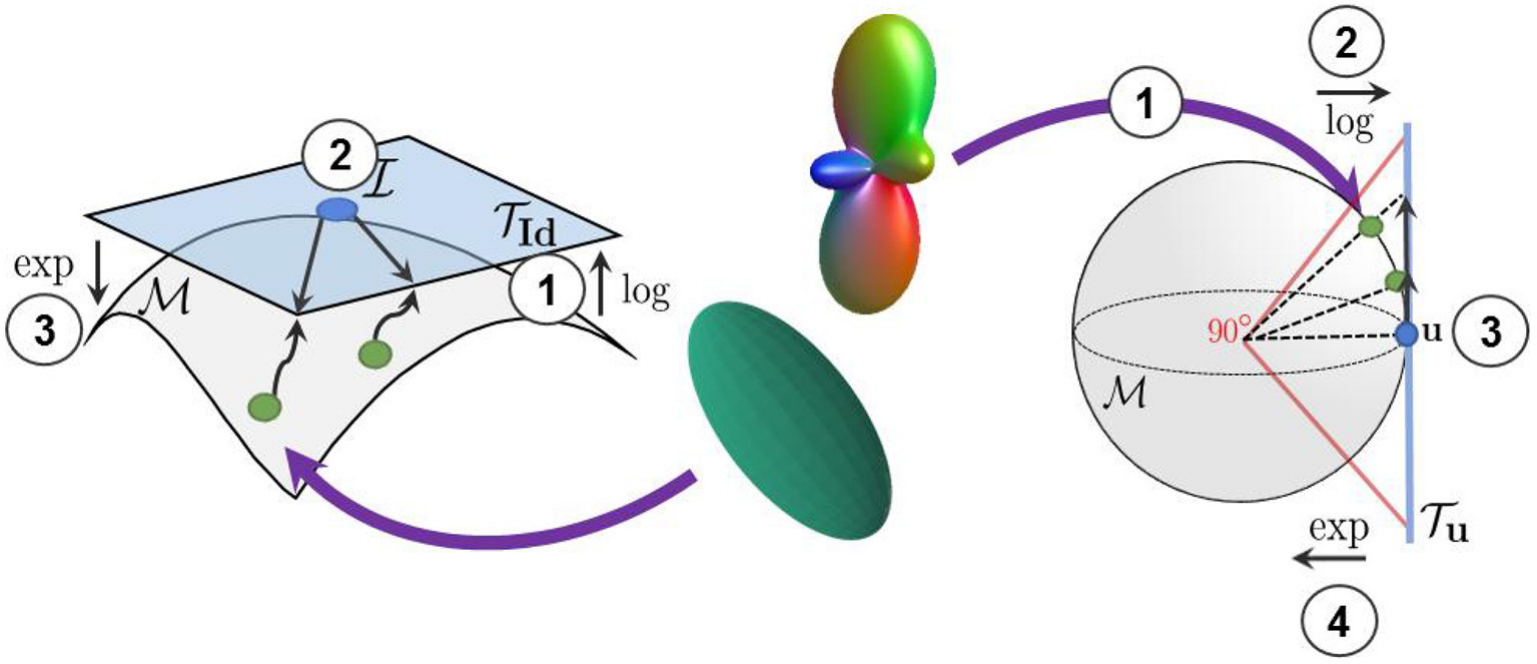
We use the log-Euclidean metrics to project the manifold-valued diffusion data to a tangent plane before processing them with Euclidean operations. (Left) The projection of diffusion tensors from the $S_{++}^{3}$ manifold to the tangent plane at the isotropic tensor $T_{\text{Id}}$. (Right) The projection of square-root re-parameterized ODF from the 𝕊^*n*^ manifold to the tangent plane at the uniform distribution $T_{\text{u}}$.

We train our network with unpaired training samples ${\left\{{\text{x}}_{i}\right\}}_{i=1}^{N}$ where **x**_*i*_ ∈ *X*_HR_ is a 3D structural image, and ${\left\{{\text{y}}_{j}\right\}}_{j=1}^{M}$ where **y**_*j*_ ∈ *Y*_LR_ is a diffusion image (i.e., a DT or ODF volume) in a lower spatial resolution. The mathematical validity of the generated diffusion is ensured by enforcing the synthesis of DT/ODF in the log-Euclidean domain using the Riemannian maps of their corresponding data manifolds as shown in Figure 3. Two discriminators *D*_*X*_ and *D*_*Y*_ evaluate the synthesized HR T1w images *G*_*X*_(**y**) and downsampled HR diffusion images, using a learned residual function F as follows $F\left(G_{Y}\left(\text{x}\right)\right)$, with regards to their real data distribution *G*_*X*_(**y**) ~ ℙ_*X*_HR__ and $F\left(G_{Y}\left(\text{x}\right)\right)\sim {\mathbb{P}}_{\log\left(Y_{\text{LR}}\right)}$. By combining pixel-wise reconstruction losses and higher-level adversarial feedback in a single objective, our MA-CycleGAN is able to exploit the local tissue information and global geometry of high-resolution structural images to produce mathematically valid diffusion data with a higher level of detail.

In the following sections, we detail the Riemannian frameworks embedded in our architecture for DT and ODF learning. Moreover, we frame our cycle-consistent and adversarial objectives incorporating both the exp and log maps of the aforementioned Riemannian frameworks and an up-and-down sampling strategy. Then, we present our anisotropy-based attention mechanism that helps the network to focus on meaningful fiber tracts information.

### 2.1. Riemannian framework for diffusion tensors learning

Diffusion tensors are 3 × 3 symmetric positive-definite matrices **M** that can be decomposed in a diagonal matrix Σ ∈ ℝ^3×3^ of real and positive eigenvalues $\lambda_{i}\in {\mathbb{R}}^{+}$ and a matrix **U** ∈ ℝ^3×3^ of corresponding eigenvectors ${\text{u}}_{i}\in {\mathbb{R}}^{3}$ using eigendecomposition such that **M** = **U**Σ**U**^⊤^. The eigenvector **u**_**1**_ associated with the largest eigenvalue λ_1_ of **M** represents the principal direction of diffusion and aligns with the underlying fibers population. DT-derived metrics, describing the shape of the tensor, are computed from the positive eigenvalues λ_1_ > λ_2_ > λ_3_ ∈ Σ. One of the most important DT-derived metric, fractional anisotropy, measures how far the shape of the diffusion tensor is from a sphere (i.e., how anisotropic the diffusion is). This metric is computed as follows:


(1) $$\begin{array}{l} \text{FA}=\sqrt{\frac{1}{2}}\frac{\sqrt{{\left(\lambda_{1}-\lambda_{2}\right)}^{2}+{\left(\lambda_{2}-\lambda_{3}\right)}^{2}+{\left(\lambda_{1}-\lambda_{3}\right)}^{2}}}{\sqrt{\left(\lambda_{1}^{2}+\lambda_{2}^{2}+\lambda_{3}^{2}\right)}}. \end{array}$$

Because of their SPD properties, tensors lie on a non-linear manifold denoted as


(2) $$\begin{array}{l} S_{++}^{3}=\{\text{M}\in {\mathbb{R}}^{3\times 3},\text{M}={\text{M}}^{⊤},{\text{x}}^{⊤}\text{M}\text{x}>0,\forall \text{x}\in {\mathbb{R}}^{3},\\ \;\left||\text{x}|{|}_{2}>0\right\} \end{array}$$

$S_{++}^{3}$ is not an Euclidean space, thus using standard Euclidean operations to process statistics on diffusion tensors can lead to undesirable effects like the swelling effect documented in Arsigny et al. (2006). Moreover, not considering the $S_{++}^{3}$ manifold while synthesizing DT with deep neural network can lead to the generation of non-SPD tensors (Gao et al., 2020). As mentioned in Pennec (2020), minimizing an Euclidean metric in the space of SPD matrices using algorithms like gradient descent can easily lead to non-SPD matrices. Indeed, in the Euclidean domain, non-SPD matrices stand at a finite distance from SPD matrices and thus, are reachable in a finite number of optimization steps. Such non-SPD tensors are physically incorrect and must be avoided. To accurately process diffusion tensors, Arsigny et al. (2006) proposed a log-Euclidean metric that enables the convenient computation of geodesic distance between DT and that push at an infinite distance non-SPD tensors. Consequently, optimizing in the space of this log-Euclidean metric (i.e., in the log-Euclidean domain) guarantees that non-SPD tensors could never be generated in a finite amount of optimization steps.

#### 2.1.1. Log-Euclidean metric

Diffusion tensor matrices are well defined in the log-Euclidean metric, where a matrix logarithm and exponential can be conveniently processed in a metric and can always be mapped back to a valid symmetric diffusion tensor (Arsigny et al., 2006). Let **M** = **UΣU**^⊤^ be the eigendecomposition of a symmetric matrix **M**. The computation of the logarithm and the exponential of a tensor noted as log_Id_ and exp_Id_ are defined as follows:


(3) $$\begin{array}{l} \forall \text{M}\in S_{++}^{3},\;\log_{\text{Id}}\left(\text{M}\right)=\;\text{U}\log\left(\Sigma \right){\text{U}}^{⊤}\in T_{\text{Id}} \end{array}$$

(4) $$\begin{array}{l} \forall \text{S}\in T_{\text{Id}},\;\exp_{\text{Id}}\left(\text{S}\right)=\;\text{U}\exp\left(\Sigma \right){\text{U}}^{⊤}\in S_{++}^{3} \end{array}$$

Using these definitions, the geodesic distance between two points on the $S_{++}^{3}$ manifold, **P**_1_ and **P**_2_, can then be expressed as


(5) $$\begin{array}{l} dist\left({\text{P}}_{1},{\text{P}}_{2}\right)=||\log_{\text{Id}}\left({\text{P}}_{1}\right)-\log_{\text{Id}}\left({\text{P}}_{2}\right)|{|}_{2}. \end{array}$$

For more details about the definition of the matrix backpropagation used to train our network, the reader is referred to Ionescu et al. (2015).

### 2.2. Riemannian framework for ODF learning

Orientation distribution functions, here represented as **p**(**s**), are probability density functions modeling the diffusion of water molecules at any point **s** on the 2-sphere 𝕊^2^. The space P of such PDFs forms the set


(6) $$\begin{array}{l} P=\{\text{p}:{𝕊}^{2}\to {\mathbb{R}}^{+}|\forall \text{s}\in {𝕊}^{2},\text{p}\left(\text{s}\right)\geq 0;\int_{\text{s}\in {𝕊}^{2}}\text{p}\left(\text{s}\right)d\text{s}=1\} \end{array}$$

The constrained function space P is not a vector space but a nonlinear differentiable manifold that, just like the aforementioned $S_{++}^{3}$ manifold, needs to be equipped with an efficient Riemannian metric to accurately process statistics on it (Srivastava et al., 2007). Fortunately, PDFs can be re-parameterized in multiple ways leading to known manifolds with closed-form and computationally-efficient Riemannian operations. The square-root re-parameterization of PDFs is a particularly convenient one as it results in a unit Hilbert sphere manifold with an 𝕃^2^ metric (Srivastava et al., 2007).

#### 2.2.1. Square root re-parameterization of ODF

With the help of the square-root re-parameterization of ODF $\psi \left(\text{s}\right)=\sqrt{\text{p}\left(\text{s}\right)},\forall \text{s}\in {𝕊}^{2}$, the space ***ψ*** can be viewed as the positive orthant of a unit Hilbert sphere:


(7) $$\begin{array}{l} \psi =\{\psi :{𝕊}^{2}\to {\mathbb{R}}^{+}|\forall \text{s}\in {𝕊}^{2},\psi \left(\text{s}\right)\geq 0;\int_{\text{s}\in {𝕊}^{2}}\psi^{2}\left(\text{s}\right)d\text{s}=1\} \end{array}$$

where the geodesic, exponential and logarithm maps are defined in a closed form. Following Descoteaux et al. (2007) and Cheng et al. (2009), ψ(**s**) is further represented in a compact way using a spherical harmonic basis, as follows:


(8) $$\begin{array}{l} \psi \left(\text{s}\right)=\overset{K}{\underset{i=1}{\sum }}c_{i}{\text{B}}_{i}\left(\text{s}\right) \end{array}$$

Here *K* is the number of orthonormal basis functions used to represent ψ(**s**) and {_**B**_*i*_}*i*∈*K*_ is the set of spherical harmonic basis functions as in Descoteaux et al. (2007). From this parametric representation, an efficient log-Euclidean framework has been proposed in Cheng et al. (2009) and is presented in Section 2.2.2. Similarly to the FA of the diffusion tensor model, the generalized fractional anisotropy (GFA) (Tuch, 2004) of the ODF can be computed from the spherical harmonic coefficients as in Equation (9) below:


(9) $$\begin{array}{l} \text{GFA}=\sqrt{1-\frac{\left(c_{0}^{0}\right)}{\sum_{k=0}^{L}\sum_{m=-k}^{k}{\left(c_{k}^{m}\right)}^{2}}}. \end{array}$$

Here, the GFA measure how far is the ODF from the uniform distribution.

#### 2.2.2. Log-Euclidean metric

Given the parametric representation of ψ(**s**) in Equation (8), the square root of any ODF can be expressed by its Riemannian coordinate $\text{c}={\left(c_{1},c_{2},\ldots ,c_{K}\right)}^{⊤}$ and gives the probability family *PF*_*K*_:


(10) $$\begin{array}{l} PF_{K}=\{p\left(\text{s}|\text{c}\right)=(\overset{K}{\underset{i=1}{\sum }}c_{i}{\text{B}}_{i}\left(\text{s}\right){)}^{2}:\int_{\text{s}}p\left(\text{s}|\text{c}\right)d\text{s}=\overset{K}{\underset{i=1}{\sum }}c_{i}^{2}=1,\\ \;=\overset{K}{\underset{i=1}{\sum }}c_{i}{\text{B}}_{i}\left(\text{s}\right)\geq 0,\forall \text{s}\in {𝕊}^{2}\}. \end{array}$$

Following Equation (10), the parameter space *PS*_*K*_ can be defined as


(11) $$\begin{array}{l} PS_{K}=\{\text{c}\;|||\text{c}||=\overset{K}{\underset{i=1}{\sum }}c_{i}^{2}=1,\;\overset{K}{\underset{i=1}{\sum }}c_{i}{\text{B}}_{i}\left(\text{s}\right)\geq 0,\forall \text{s}\in {𝕊}^{2}\} \end{array}$$

which is also a subset of the sphere manifold 𝕊^*K*−1^. The sphere, being a simple and well-studied manifold, makes the log-Euclidean framework for ODFs computation straight-forward and efficient as seen in Equations (12) and (13) below:


(12) $$\begin{array}{l} \forall \text{c}\in PS_{K}\subset S^{k-1},\log_{\text{u}}\left(\text{c}\right)=\frac{\text{c }-\text{ u}\text{ cosΨ}}{||\text{c }-\text{ u}\text{ cosΨ}|{|}_{2}}\text{Ψ},\\ \;\text{where Ψ}=\text{arcos}\left(\langle \text{u}|\text{c}\rangle \right) \end{array}$$

(13) $$\begin{array}{l} \forall {\text{v}}_{\text{c}}\in T_{\text{u}},\exp_{\text{u}}\left({\text{v}}_{\text{c}}\right)=\text{u }\text{cos Ψ}+\frac{{\text{v}}_{\text{c}}}{||{\text{v}}_{\text{c}}|{|}_{2}}\text{sin Ψ},\\ \;\text{where Ψ}=||{\text{v}}_{\text{c}}|{|}_{2}. \end{array}$$

Here, **u** is the uniform orientation distribution function defined as **u** = (1, 0, …, 0). We use the maps in Equations (12) and (13) to accurately learn ODFs and ensure their validity throughout the training process. Furthermore, the log-Euclidean framework offers a simple geodesic estimation between two parameterized ODFs *p*(·|**c**) and *p*(·|**c**′) as follows:


(14) $$\begin{array}{l} dist\left(p\left(\cdot |\text{c}\right),p\left(\cdot |{\text{c}}^{\prime }\right)\right)=||\log_{\text{u}}\left(\text{c}\right)-\log_{\text{u}}\left({\text{c}}^{\prime }\right)|{|}_{2}. \end{array}$$

### 2.3. Adversarial training

In a standard GAN setup as in Goodfellow et al. (2014), a generator network *G* tries to generate samples so close to the true data distribution that a discriminator network *D* is unable to distinguish between real and fake examples. Following the CycleGAN architecture, our method uses two generators and two discriminators denoted as *G*_*X*_, *G*_*Y*_, *D*_*X*_, and *D*_*Y*_. Here, *G*_*X*_ takes as input a batch of LR diffusion volumes in the log-Euclidean domain that have been upsampled, using trilinear interpolation, to the spatial resolution of the target HR T1w images (i.e., 0.7 mm^3^ voxels). *G*_*X*_ tries to fool *D*_*X*_ by generating realistic high-resolution T1w volumes that are indistinguishable from real HR T1w images. Similarly, *G*_*Y*_ takes HR T1w volumes as input and tries to generate plausible diffusion volumes in the log-Euclidean domain at the same level of details.

**Figure 4:**
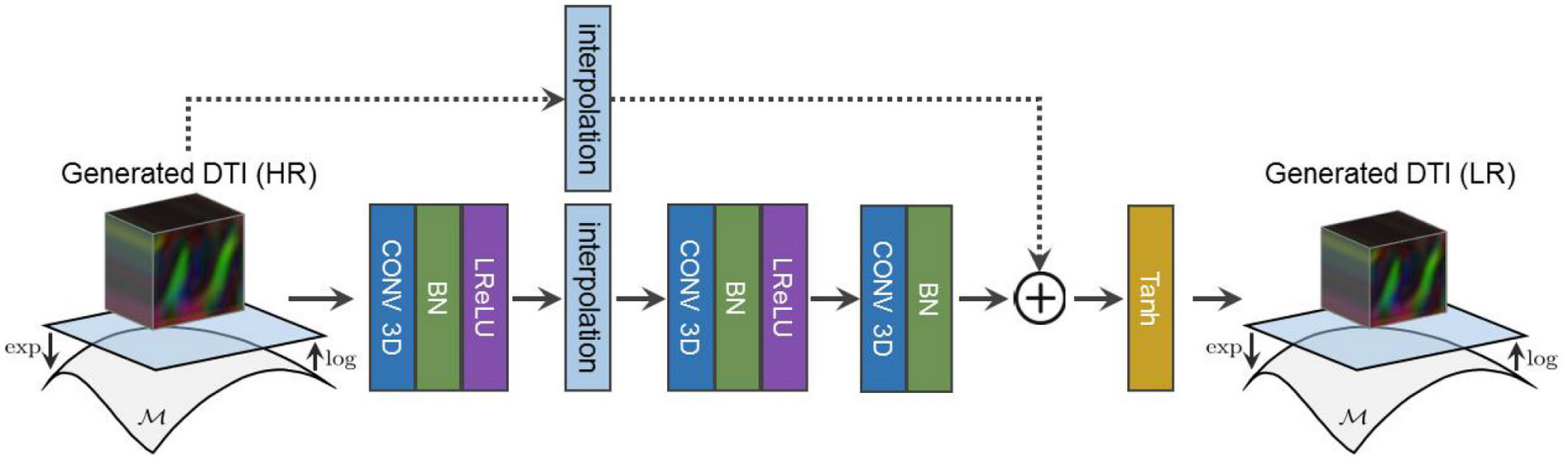
The architecture of our residual downsampling function F used to reduce the resolution of our synthesized HR diffusion before *D*_*Y*_ assessment.

Because we only have access to real LR diffusion data, the synthesized HR diffusion is downsampled using a learned residual function F prior to *D*_*Y*_'s assessment as shown in Figure 4. Our adversarial objectives follow the LSGAN formulation in Mao et al. (2017) and are expressed as follows:


(15) $$\begin{array}{l} \underset{D_{Y}}{\min}L_{\text{LSGAN}}\left(G_{Y},D_{Y},X_{\text{HR}},Y_{\text{LR}}\right)\;=\\ \;\frac{1}{2}{𝔼}_{\text{y}\sim {\mathbb{P}}_{Y_{\text{LR}}}}\left[{\left(D_{Y}\left(\log\left(\text{y}\right)\right)-1\right)}^{2}\right]\\ \;+\frac{1}{2}{𝔼}_{\text{x}\sim {\mathbb{P}}_{X_{\text{HR}}}}\left[{\left(D_{Y}\left(F\left(G_{Y}\left(\text{x}\right)\right)\right)\right)}^{2}\right] \end{array}$$

(16) $$\begin{array}{l} \underset{D_{X}}{\min}L_{\text{LSGAN}}\left(G_{X},D_{X},Y_{\text{LR}},X_{\text{HR}}\right)\;=\\ \;\frac{1}{2}{𝔼}_{\text{x}\sim {\mathbb{P}}_{X_{\text{HR}}}}\left[{\left(D_{X}\left(\text{x}\right)-1\right)}^{2}\right]\\ \;+\frac{1}{2}{𝔼}_{\text{y}\sim {\mathbb{P}}_{Y_{\text{LR}}}}\left[{\left(D_{X}\left(G_{X}\left(↑\log\left(\text{y}\right)\right)\right)\right)}^{2}\right] \end{array}$$

where ↑ represents trilinear upsampling and log is the logarithm map defined in either Equation (3) or Equation (12) depending on the diffusion model that is synthesized. It should be noted that both the upsampling of the real data and the discriminator evaluation of the generated diffusion information are performed in the log-Euclidean domain to account for the underlying data manifold.

### 2.4. Cycle-consistency loss

The adversarial losses alone are not sufficient to drive the generation of HR diffusion. Indeed, *D*_*Y*_ only evaluates downsampled diffusion volumes and, thus, cannot help *G*_*Y*_ improving beyond a certain precision level. Therefore, the cycle-consistency loss denoted in Equation (17) not only helps ensuring the structural coherence of the synthesized images across modalities, but also provides important high-resolution gradients to train *G*_*Y*_.

Our cycle-consistency loss is three-fold: (1) the error between the original HR structural volume **x** and the reconstructed volume *G*_*X*_(*G*_*Y*_(**x**)), (2) the error between the upsampled diffusion ↑log(**y**) and its HR reconstruction *G*_*Y*_(*G*_*X*_(↑log(**y**))), and (3) the error between the original LR diffusion **y** and the downsampled recovered volume $F\left(G_{Y}(G_{X}\left(↑\log\left(\text{y}\right)\right))\right)$. Combining these in a single loss gives


(17) $$\begin{array}{l} L_{\text{cyc}}\left(G_{Y},G_{X}\right)\;=\;\lambda_{{\text{cyc}}_{X}}\underset{\text{Forward Cycle HR}}{\underset{︸}{{𝔼}_{\text{x}\sim {\mathbb{P}}_{X_{\text{HR}}}}\left[||G_{X}\left(G_{Y}\left(\text{x}\right)\right)-\text{x}|{|}_{1}\right]}}\\ \;+\;\frac{1}{2}\lambda_{{\text{cyc}}_{Y}}\underset{\text{Backward Cycle HR}}{\underset{︸}{{𝔼}_{\text{y}\sim {\mathbb{P}}_{Y_{\text{LR}}}}\left[||G_{Y}(G_{X}\left(↑\log\left(\text{y}\right)\right))-↑\log\left(\text{y}\right)|{|}_{1}\right]}}\\ \;+\;\frac{1}{2}\lambda_{{\text{cyc}}_{Y}}\underset{\text{Backward Cycle LR}}{\underset{︸}{{𝔼}_{\text{y}\sim {\mathbb{P}}_{Y_{\text{LR}}}}\left[||F(G_{Y}(G_{X}\left(↑\log\left(\text{y}\right)\right)))-\log\left(\text{y}\right)|{|}_{1}\right]}} \end{array}$$

**Figure 5:**
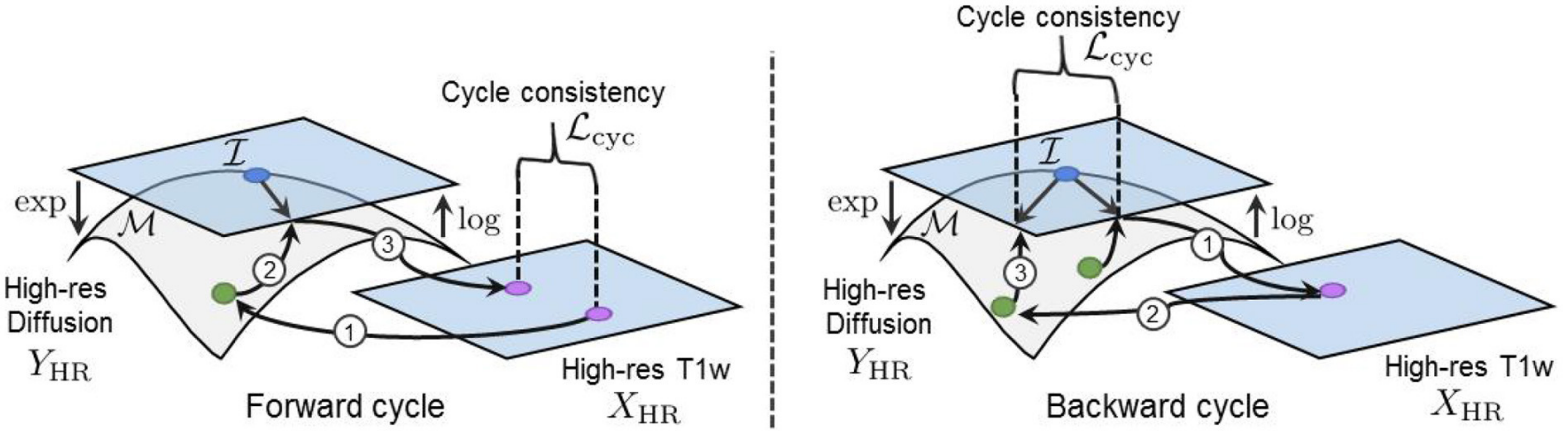
(Left) Our forward cycle: A T1w image is translated to a high-resolution DT on the $S_{++}^{3}$ manifold and back to the T1w domain where the cycle consistent loss is computed. (Right) Our backward cycle: An upsampled DT on $T_{\text{Id}}$ is translated to the T1w domain and back to $T_{\text{Id}}$ where the cycle consistent loss is computed.

We employ the ℓ_1_ norm in Equation (17) to measure both the forward and backward cycle reconstruction errors, as it is less sensitive to large errors than the ℓ_2_ norm (Zhao et al., 2016). Again, the log map is used to project the generated and the real manifold-valued data onto a tangent plane before computing the cycle-consistency loss. This projection enables the accurate computation of the distance between each DT/ODF of the synthesized and real diffusion volumes and ensures the mathematical validity of the synthesized images during training. Indeed, a DT/ODF in the log-Euclidean domain can always be mapped back to a mathematically valid DT/ODF using the Riemannian exp(·) map of their respective Riemannian framework (see Sections 2.1 and 2.2). Furthermore, two parameters λ_cyc_*X*__ and λ_cyc_*Y*__ control the contribution of both cycles and have been empirically tuned to compensate for the difference in the scale of the different terms of the loss (i.e., more weight was given to the smaller loss terms). Full cycles, including the manifold mappings and the loss computation in the tangent plane, are illustrated in Figure 5.

### 2.5. Image prior regularization

By using a cycle-consistency loss in both directions, the CycleGAN model is able to learn a bijective mapping between two domains using unpaired examples (Zhu et al., 2017). However, for many cross-domain translation problems, the solution space is extremely large and the model does not necessarily converge to a solution that satisfies important domain-specific properties (Lu et al., 2019). This is problematic, especially in the case of medical images synthesis where the generated images must not only be realistic from the discriminator's point of view, but also be faithful to expected results of the downstream tasks and known anatomical properties. Thus, to ensure the model's convergence toward plausible solutions, we introduce a prior loss as follows:


(18) $$\begin{array}{l} L_{\text{prior}}\left(G_{Y},G_{X}\right)\;=\;\lambda_{{\text{prior}}_{Y}}{𝔼}_{\text{x}\sim {\mathbb{P}}_{X_{\text{HR}}},\text{y}\sim {\mathbb{P}}_{Y_{\text{LR}}}}\\ \;\left[||G_{Y}\left({\text{x}}_{i}\right)-↑\log\left({\text{y}}_{i}\right)|{|}_{1}\right]\\ \;+\;\lambda_{{\text{prior}}_{X}}{𝔼}_{\text{x}\sim {\mathbb{P}}_{X_{\text{HR}}},\text{y}\sim {\mathbb{P}}_{Y_{\text{LR}}}}\left[||G_{X}\left(↑\log\left({\text{y}}_{i}\right)\right)-{\text{x}}_{i}|{|}_{1}\right] \end{array}$$

where **x**_*i*_ and **y**_*i*_ are paired volumes (i.e., HR T1w volumes and aligned and upsampled diffusion volumes of the same subjects) taken from a limited number of subjects. With this loss, the super-resolved diffusion stays close to the real upsampled diffusion while integrating high-frequency elements from the HR structural images.

### 2.6. Diffusion anisotropy weighted loss

**Figure 6:**
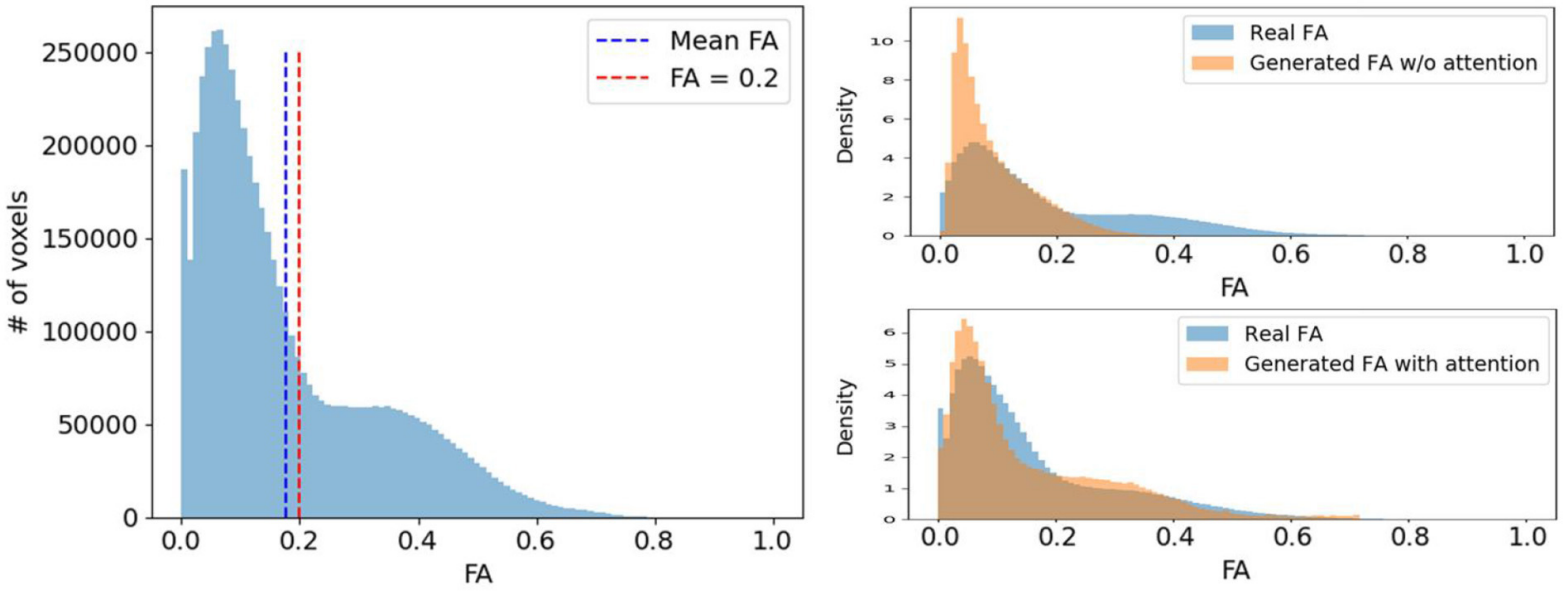
(Left) A typical FA distribution of a randomly selected subject. (Right) The comparison of the real FA (blue) and the generated FA (orange) distribution density of the same subject with and without diffusion anisotropy weighted loss.

Voxels expressing fiber tracts information typically have higher FA values than those representing other tissues like gray matter (GM) or cerebrospinal fluid (CSF). Therefore, we would like our diffusion synthesis method to be particularly accurate in regions with higher fractional anisotropy. However, as seen in Figure 6, voxels with high FA are underrepresented compared to those with lower values. Consequently, this imbalance problem drives the network's generation toward diffusion with FA close to the mean. To alleviate this issue, we weight the diffusion error in $L_{\text{cyc}}$ and $L_{\text{prior}}$ by the FA/GFA of the target volume at every voxel. The benefit of such mechanism can be observed on the density plots at the bottom of Figure 6 which clearly exhibit a more faithful FA distribution when using the proposed diffusion anisotropy weighting scheme.

### 2.7. Full objective

Combining all loss terms, our full objective function is given by


(19) $$\begin{array}{l} L\;=\;-L_{\text{LSGAN}}\left(G_{X},D_{X},Y_{\text{LR}},X_{\text{HR}}\right)\\ \;-L_{\text{LSGAN}}\left(G_{Y},D_{Y},X_{\text{HR}},Y_{\text{LR}}\right)\\ \;+L_{\text{cyc}}\left(G_{Y},G_{X}\right)+L_{\text{prior}}\left(G_{Y},G_{X}\right) \end{array}$$

As in standard adversarial learning approaches, we train the generators and discriminators concurrently by solving a mini-max problem:


(20) $$\begin{array}{l} {G_{X}}^{*},{G_{Y}}^{*}=\arg\underset{G_{X},G_{Y}}{\min}\underset{D_{X},D_{Y}}{\max}L\left(G_{X},G_{Y},D_{X},D_{Y}\right) \end{array}$$

Hence, Equation (19) combines the error feedback from both the HR structural images and the LR diffusion in an adversarial and voxel-wise manner.

## 3. Results

### 3.1. Data and pre-processing

We employ the T1w and diffusion MRI data of 1,065 subjects from the HCP1200 release of the Human Connectome Project (Van Essen et al., 2013) to evaluate our manifold-aware CycleGAN. The T1w (0.7 mm^3^ voxels, FOV = 224 mm, matrix = 320, 256 sagittal slices in a single slab) and diffusion (1.25 mm^3^ voxels, sequence = Spin-echo EPI, repetition time (TR) = 5,520 ms, echo time (TE) = 89.5 ms) data were acquired with a Siemens Skyra 3T scanner (Sotiropoulos et al., 2013) and minimally processed following (Glasser et al., 2013).

Diffusion tensors were fitted using the DSI Studio toolbox software (Jiang et al., 2006) and the dODFs estimated using the constant solid angle (CSA) method (Aganj et al., 2010) from the DIPY library (Garyfallidis et al., 2014). Diffusion ODFs were then re-parameterized following Section 2.2.1 and further estimated using 4th order spherical harmonics (Descoteaux et al., 2007). Both the DT and the ODF volumes were transformed to the log-Euclidean domain using their respective Riemannian framework described in Section 2. Once in the log-Euclidean domain, these volumes were upsampled to the T1w spatial resolution using trilinear interpolation and aligned to their corresponding HR T1w images. In experiments, we consider these upsampled and aligned diffusion volumes as the “ground truth” HR diffusion. Indeed, we do not have access to real diffusion volumes in the same spatial resolution as the T1w images (i.e., 0.7 mm^3^ voxels) therefore, we consider the upsampled diffusion volumes as the gold standard. Even though the upsampled diffusion volumes are not as informative as real HR diffusion volumes, they still represent a valid and valuable target objective. For instance, the upsampling of diffusion volumes is nowadays a default step in MRI processing tools such as in TractoFlow (Theaud et al., 2020) and MRtrix3 (Tournier et al., 2019). The T1w images have been rescaled to the [0,1] range by min-max normalization. Finally, both the structural and the diffusion volumes, in high and low-resolution, were decomposed in overlapping patches of 32^3^ and 18^3^ voxels, respectively. Volumes are processed patch-wise by our model for two important reasons: (1) limiting the memory required by the model to compute network activations and outputs, and (2) increasing the amount of training examples.

### 3.2. Implementation details

Our two generator networks, *G*_*X*_ and *G*_*Y*_, follow the U-Net implementation in Çiçek et al. (2016) where the last activation layer has been replaced to fit the different output data scale. In all the experiments, we used a sigmoid in *G*_*X*_ to generate T1w images within the [0, 1] range. For the generation of diffusion, the last activation function of *G*_*Y*_ varies depending on the generated diffusion model. For the generation of DT, we used a hard hyperbolic tangent function and for the generation of ODF, a tanh activation.

Moreover, we set the amount of input and output channels of our networks according to our input and output data shape. The DT inputs are of shape 9 × 32 × 32 × 32, where the nine channels represent the flattened 3 × 3 diffusion tensors at every voxel. The ODF inputs are of shape *C* × 32 × 32 × 32 where C depends on the order of the spherical harmonic basis that is used to represent them. Due to limitations on computational resources, we estimated the diffusion ODFs using 4th order spherical harmonics, yielding a total of *C* = 15 coefficients.

Our discriminator networks *D*_*X*_ and *D*_*Y*_ follow the SRGAN discriminator architecture in Ledig et al. (2017) where we replaced the 2D convolutions by 3D ones. Furthermore, we reduced the number of feature maps in convolution layers to 32, 64, 128, and 256 as suggested in Sánchez and Vilaplana (2018) for volumetric data. In all scenarios, *D*_*X*_ assesses T1w volumes of shape 1 × 32 × 32 × 32 while *D*_*Y*_ takes as inputs volumes of shape 9 × 18 × 18 × 18 for DT and 15 × 18 × 18 × 18 for ODFs.

### 3.3. Training setup

To train our network, we randomly selected 70% (746 subjects) of the 1,065 subjects for training, 20% (213 subjects) for validation and 10% (106 subjects) for testing. From the 746 training subjects, we kept aside 50 subjects as paired priors in Equation (18) and split the remaining 696 subjects into two groups of 348 subjects. To form our unpaired training set, we sampled 50,000 patches from the diffusion images of the first group of 348 training subjects and 50,000 patches from the T1w images of the second group of 348 training subjects. The same number of patches (50,000) was selected from our 50 subjects kept as paired priors, i.e., aligned HR T1w and upsampled diffusion. For the validation and test sets, we randomly selected 10,000 paired patches from validation subjects and 5,000 paired patches from test subjects.

We trained the networks using an Adam optimizer (Kingma and Ba, 2015) with a learning rate of 10^−4^ and beta1, beta2 values of 0.5 and 0.999. Hyper-parameters λ_prior_*X*__, λ_prior_*Y*__, λ_cyc_*X*__ and λ_cyc_*Y*__ were set to 10, 0.5, 5, and 0.25. Furthermore, we used a reduce on plateau learning rate scheduler with a patience of 10 epochs and a factor of 10. Batches of eight patches were used and the models were trained for 35 epochs (~220k steps) on an NVIDIA TITAN XP GPU with 12 GB of VRAM. All experiments were repeated three times with a different initialization seed.

### 3.4. Baselines

As mentioned before, deep learning models for the synthesis of manifold-valued data are just starting to emerge. Consequently, the number of baselines for the task of diffusion synthesis from structural imaging that require minimal adaptation to evaluate our model is limited. Nevertheless, we compare our model to the three approaches described below.

#### 3.4.1. Manifold-aware WGAN

Our first baseline is an adaptation of the manifold-aware WGAN presented in Huang et al. (2019) for the conditional generation of diffusion from structural T1w images. This method denoted as “MA-WGAN” in our results, can generate plausible manifold-valued images by incorporating the log-Euclidean maps within the network and therefore provides a natural point of comparison for our method. For this baseline, we use the same generator *G*_*Y*_ and discriminator *D*_*Y*_ as in our proposed model. The manifold mapping used in the network is changed according to the generated diffusion scheme following Section 2.

#### 3.4.2. Manifold-aware U-Net

We also compare our method to a supervised U-Net model. For this baseline, we use the same generator as in our own architecture, i.e., *G*_*Y*_ (Çiçek et al., 2016), but train it in a supervised manner with paired HR T1w and upsampled diffusion volumes in the log-Euclidean domain. Similar to the *MA-WGAN* baseline, we change the manifold mapping of the network according to the generated diffusion reconstruction scheme (DT or ODF). This baseline, denoted as “MA-U-Net” in results, helps us measure the effect of our adversarial and cycle-consistent losses.

#### 3.4.3. U-Net

Our last baseline is a standard supervised U-Net (Çiçek et al., 2016) trained with paired HR T1w images and upsampled diffusion without manifold-awareness. With this method, we aim at measuring the performance gain of our method induced by both the manifold-mapping and the use of additional unpaired samples. In addition, we validate that manifold-awareness is necessary to synthesize realistic samples strictly lying on the data manifold.

One should note that we are using the same generator network architecture (Çiçek et al., 2016) in all baselines so that they have comparable inference latency (i.e., 9.32±0.26 s) for the translation of a T1w volume to a DTI volume and 11.73 ± 0.32 s for the translation to ODF) and the exact same amount of parameters at inference (16.32 M for DTI synthesis and 16.33 M for ODF).

### 3.5. DT and ODF synthesis

We first test our network and baselines for the task of DT and diffusion ODF synthesis. In this setup, we train our network with unpaired HR T1w patches and LR diffusion patches in the log-Euclidean domain. Moreover, we use a paired training set of 50,000 patches from 50 randomly chosen subjects. The paired training set is used as prior for our method and as the training set for our baselines. Hence, both our method and baselines are trained with the same amount of paired information.

#### 3.5.1. Evaluation metrics

Three metrics were considered to quantitatively evaluate the generated diffusion. First, we use the cosine similarity to compare the principal fiber orientation of every synthesized tensor and ODF to their expected real orientation:


(21) $$\begin{array}{l} \text{similarity}\left(\text{a},\text{b}\right)=\left|\frac{\text{a}\cdot \text{b}}{\left||\text{a}||||\text{b}|\right|}\right| \end{array}$$

To retrieve the main orientation of the ODF, we first calculate the spherical coordinates at which the value of the ODF is maximum using a discretized sphere of 724 vertices. We then convert these spherical coordinates to Euclidean coordinates to obtain their principal orientation vector. For DT, the main orientation is given by the eigenvector associated with the largest eigenvalue of the DT as described in Section 2.1.

In addition, we compare the generated and real diffusion with the mean square error (MSE) between their FA/GFA. The FA and GFA are computed following Equations (1) and (9). This help evaluating the shape of the synthesized DT/ODF independently of their orientation.

Finally, we measure the mean geodesic distance using Equation (5) for DT and Equation (14) for ODF between our generated data and the real diffusion. This latter metric encode both the orientation and the shape error in a single measure.

### 3.6. Tractography

To further assess the integrity of the synthesized diffusion volumes by the proposed method, we performed whole-brain tractography on both the real and the generated data, and segmented the resulting tractograms into bundles. We then posed the tractograms generated on real data as ground truth and extracted quantitative measures from whole-brain tractograms. Likewise, we segmented bundles to better appreciate the differences between the tractograms produced using real diffusion and generated diffusion from structural inputs. In the following subsections, we describe each step of the analysis.

#### 3.6.1. Streamlines generation and bundling

Tracking was performed using the EuDX algorithm (Garyfallidis, 2013) with a step-size of 0.5 mm. A maximum angle of 60° was used between steps, using the principal direction of the diffusion tensor and maxima of ODF. Maxima were extracted from the ODF using scilpy. Seeding was done at two seeds per voxel on the whole white-matter mask, which was computed from the ground-truth T1w image using Dipy (Garyfallidis et al., 2014). Streamlines with a length below 10 mm or above 300 mm were discarded. Whole brain tractograms were then segmented using RecobundlesX (Garyfallidis et al., 2018), using 80 bundles from Yeh et al. (2018) as reference. To allow for a more robust comparison, and because initial streamline points placement depends on randomness which may have an impact on the reconstructed streamlines, tracking was performed five times with different random seeds on each volume.

#### 3.6.2. Streamlines assessment

The similarity between the streamlines reconstructed from the real and generated diffusion volumes is assessed using three measures: streamline length, bundle volume and bundle shape. First, we compare the streamline length (in mm) between all real and synthesized whole-brain tractograms, as well as each segmented bundle. We also compare the volume occupied by the whole-brain tractograms and bundles in a voxel-wise matter. Furthermore, the shape similarity of reconstructed tractograms is measured by their voxel-wise Dice, overlap (OL) and overreach (OR). The OL, defined as


(22) $$\begin{array}{l} \text{OL}=\frac{\left|B\cap A\right|}{\left|A\right|}, \end{array}$$

where *A*, *B* are binary bundle masks, quantifies how much the volume of bundle *A* is reconstructed by bundle *B*. The OR, expressed as


(23) $$\begin{array}{l} \text{OR}=\frac{\left|B\cup A|-|B\cap A\right|}{\left|A\right|}, \end{array}$$

evaluates how much of bundle *B* goes over bundle *A*. Segmented bundles were merged to allow for pairwise comparison between real and generated data. Merged bundles with fewer than 100 streamlines were discarded from the analysis. To evaluate the overall reconstruction quality, we also report Dice, OL and OR between the reconstructed tractograms from the real and the generated diffusion.

**Figure 7:**
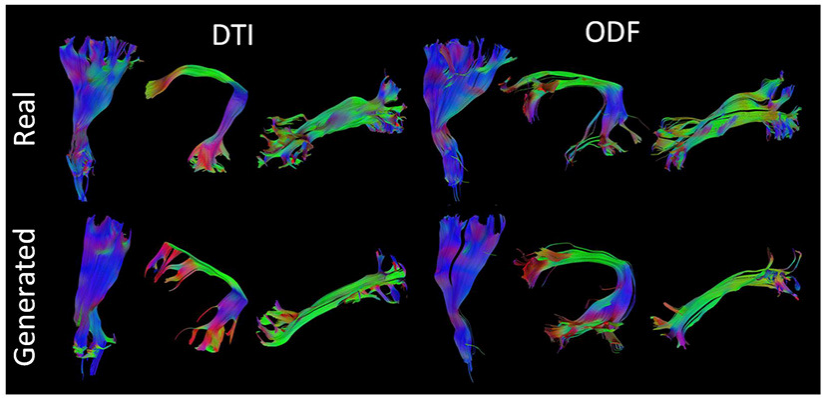
Visualization of three segmented bundles from real and synthesized tractograms. (Left) Left Corticospinal Tract (CST), Arcuate Fasciculus (AF), and Inferior Longitudinal Fasciculus (ILF) segmented from real and generated DT data. (Right) Left CST, AF, and ILF segmented from real and synthesized ODF data.

Figure 7 presents some of the reconstructed bundles from real and synthesized data.

### 3.7. Diffusion synthesis analysis

As reported in Table 1, our model yields a mean cosine similarity of 0.8648 ± 5.5 × 10^−3^ and 0.8846 ± 4.4 × 10^−3^ in voxels with FA ≥ 0.2 for DT and ODF, respectively. In voxels with FA ≥ 0.5, a mean cosine similarity of 0.9167 ± 3.2 × 10^−3^ is reached for DT and 0.9425 ± 1.4 × 10^−4^ for ODF. This corresponds to FA MSE values of 0.0089 ± 1.5 × 10^−3^ and 0.0159 ± 1.4 × 10^−4^ for DT and GFA MSE values of 0.0229 ± 2.8 × 10^−4^ and 0.0614 ± 1.3 × 10^−3^ for ODF.

**Table 1:** The fractional anisotropy mean squared error (FA MSE), geodesic, and cosine similarity between the principal orientations obtained by the different compared methods when trained with 50 paired subjects.

	**Method**	**No. of parameters**	**FA** ^*^ **MSE**		**Geodesic**		**Cosine similarity**	
			FA^*^≥0.2	FA^*^≥0.5	FA^*^≥0.2	FA^*^≥0.5	FA^*^≥0.2	FA^*^≥0.5
DT	U-Net	16.32 M	0.0100	0.0207	2.2559	2.5457	0.8266	0.8795
	MA-WGAN	17.19 M	0.0294	0.0781	0.7791	1.046	0.5724	0.6246
	MA-U-Net	16.32 M	**0.0088**	0.0184	0.3947	0.5415	0.8288	0.8863
	**Ours**	34.36 M	0.0089	**0.0159**	**0.3531**	**0.4809**	**0.8648**	**0.9167**
ODF	U-Net	16.33M	0.0220	**0.0559**	0.3639	0.6299	0.8702	0.9045
	MA-WGAN	17.21 M	0.0443	0.1173	0.5351	0.9121	0.6823	0.7278
	MA-U-Net	16.33 M	**0.0219**	0.0563	0.3611	0.6249	0.8710	0.9047
	**Ours**	34.4 M	0.0229	0.06145	**0.3467**	**0.6238**	**0.8846**	**0.9425**

Since the metrics are more relevant in regions that typically encode fibers information, we report them at increasing FA thresholds of 0.2 and 0.5.

* FA is used for DT and GFA for ODF. The bold values represent the best results.

As a point of comparison, we also give in Table 1 the performance of our baselines when they are trained with the same 50 paired subjects. As can be seen, the proposed model obtains the highest performance for all metrics when trained on DT images, as well as better geodesic and fiber orientation estimation than baselines when trained on ODF.

Compared to the fully-supervised U-Net, which does not enforce manifold consistency on the output, our model improves FA MSE by 23.14%, mean geodesic distance by 81.11% and mean cosine similarity by 4.23% for regions with FA ≥ 0.5 for DT. This shows the benefit of imposing manifold-awareness constraints on the network's output. Without these constraints, U-Net generates an average of 3,843.5 ± 481.22 non-SPD tensors and 1,559.7 ± 122.3 non-PDF ODF. Our model also provides statistically better performance, for both DT and ODF data, on the mean cosine similarity and mean geodesic distance compared to MA-U-Net (paired *t*-test *p* < 0.05), that does not include cycle-consistency and is only trained with paired data.

Improvements are particularly important for voxels with FA ≥ 0.5, where our method obtains a 3.43% higher mean cosine similarity, 11.19% lower geodesic and 13.58% better FA MSE for DT and 4.18% higher mean cosine similarity for ODF. This demonstrates the impact of our anisotropy-weighted loss described in Section 2.6, which gives more importance to voxels with higher anisotropy values.

**Figure 8:**
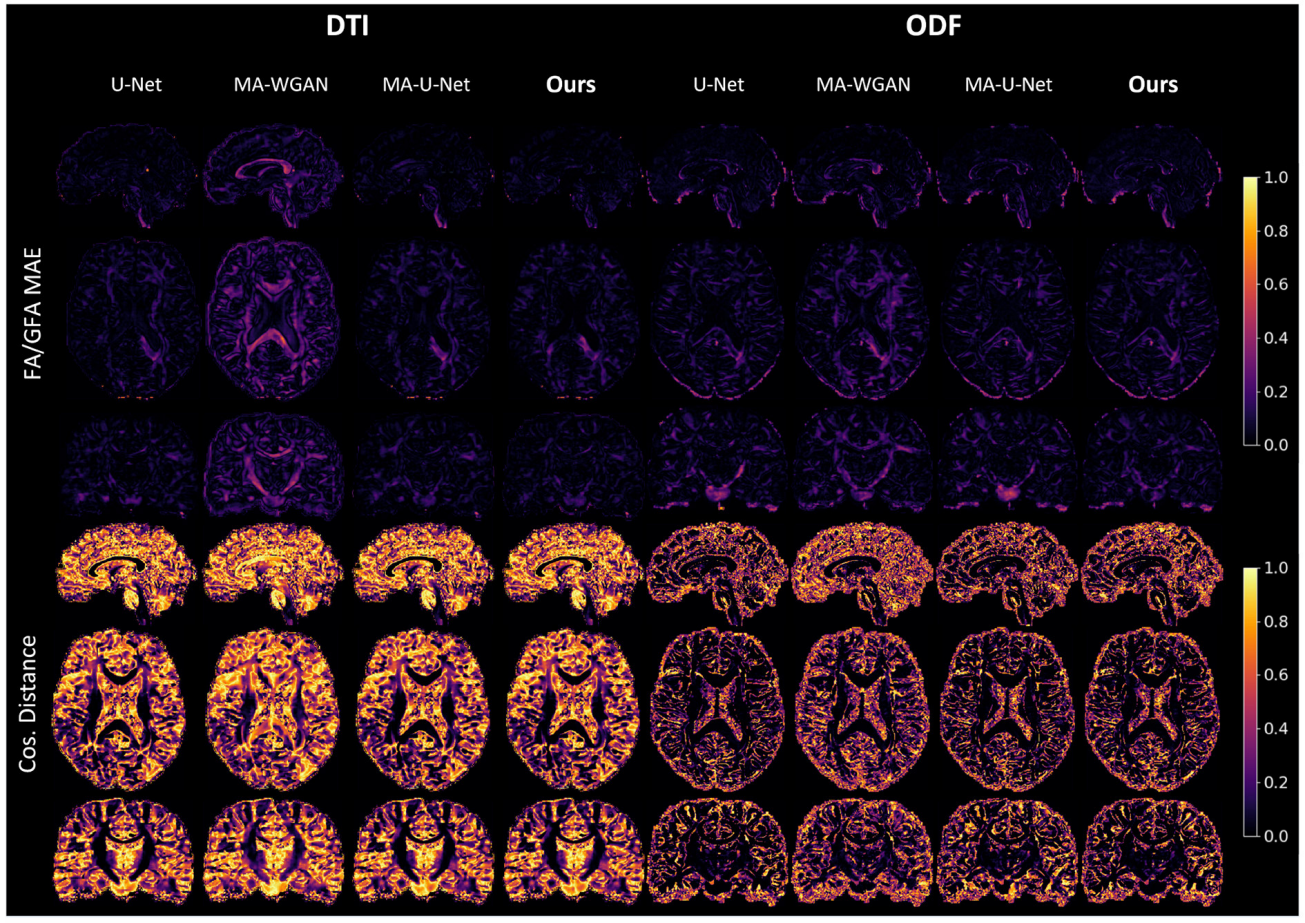
FA MAE and cosine distance on the sagittal, coronal and axial slices between the generated HR diffusion by the compared methods and the interpolated ground-truth of a random test subject. A lower FA MAE indicates a more faithful estimation of the DT/ODF shape, while a lower cos. distance indicates a better estimation of their principal orientation.

Our results can be further appreciated in Figure 8, where we report the FA and cosine error yielded by our method and baselines on a sagittal, axial and coronal slice of a randomly chosen test subject. We see that our network is able to recover most of the fibers orientation, especially in regions with typically higher FA/GFA like the corpus callosum. Moreover, the estimated FA/GFA is generally faithful to the real data except in the corticospinal tract where the error is higher.

**Figure 9:**
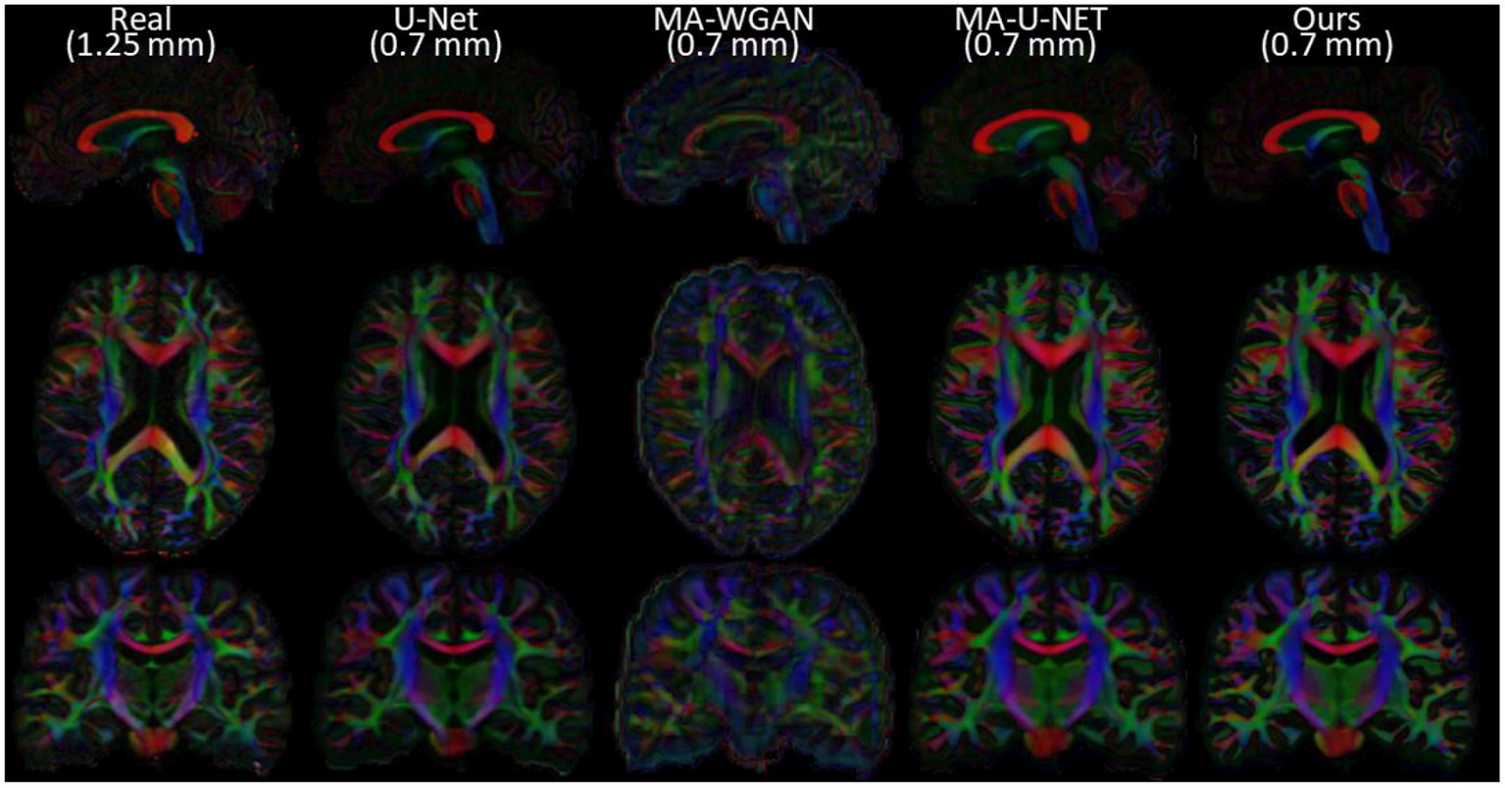
Comparison of the color FA of the generated diffusion of compared methods on a sagittal, axial, and coronal slice of a random test subject.

**Figure 10:**
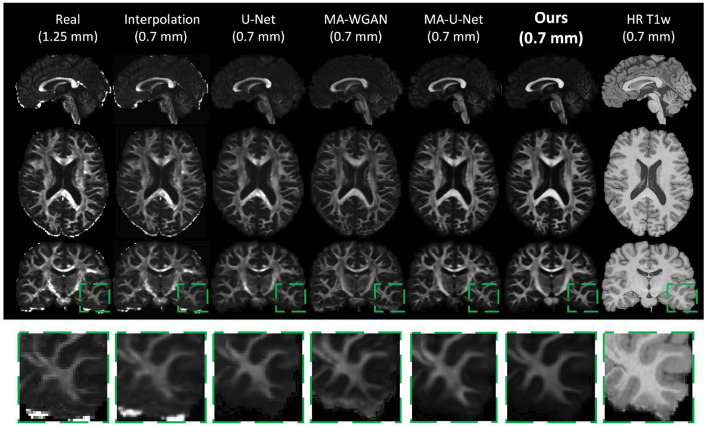
GFA of the real low-resolution diffusion (first column), the real upsampled diffusion (second column) and the generated diffusion of the compared methods. One can notice the ability of our network to recover fine anatomical details that are clearly visible in the HR T1w image but not in the original diffusion.

**Figure 11:**
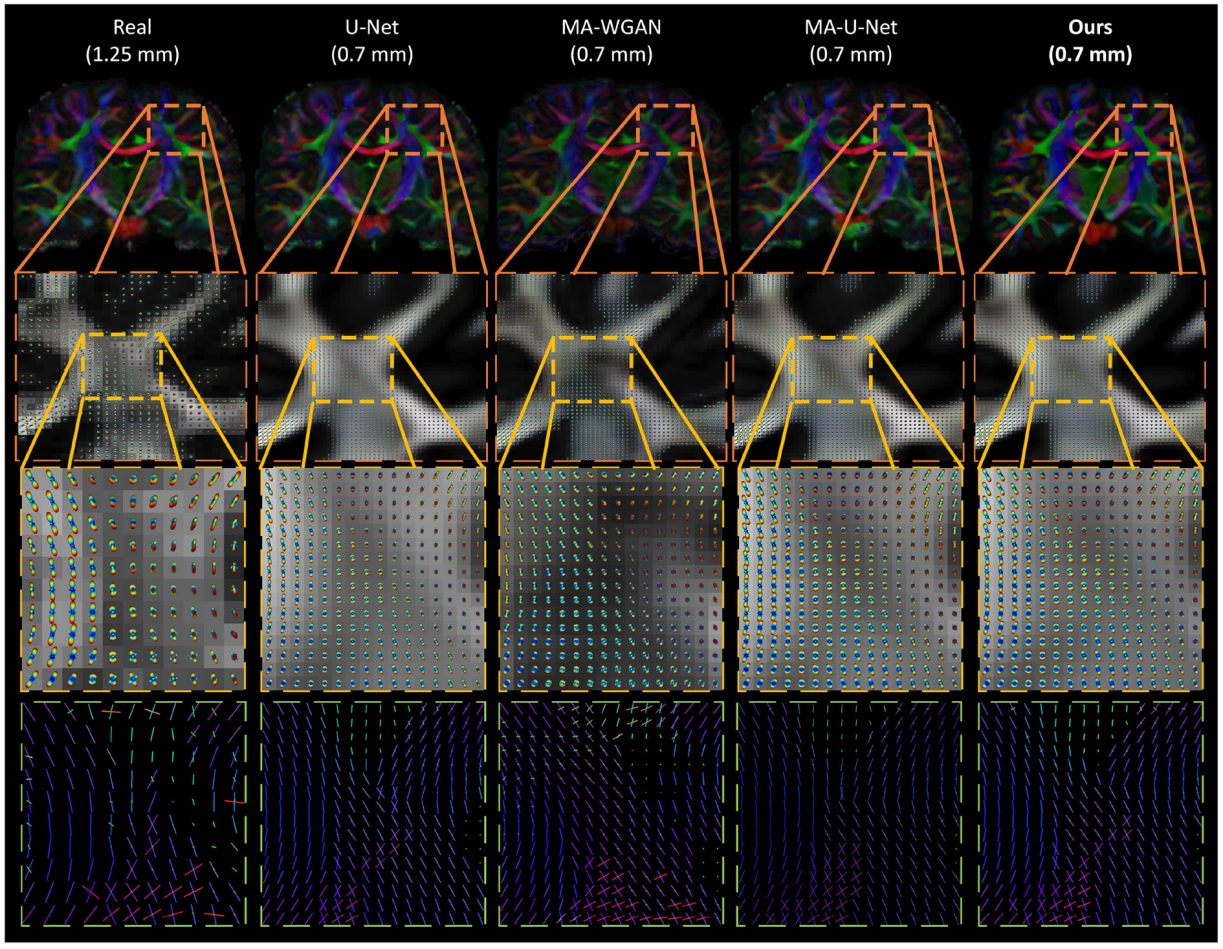
Qualitative comparison of the generated ODF by the compared methods. (First row) The color encoded GFA of a coronal slice of a test subject. (Second row) The generated ODF and GFA in a region with crossing fibers. (Third row) A close-up look of the generated ODF and GFA. (Fourth row) A close-up look at the ODF peaks. While all the compared methods are able to recover some crossing fibers, MA-WGAN seems to generate more of them, most of which are not present in the real diffusion ground-truth.

Finally, as a qualitative evaluation, we compare in Figure 9 the generated color encoded FA of compared methods. From this figure, we can see how our cycle-consistent and prior losses help our model converging toward plausible solutions better than baselines, especially compared to the MA-WGAN that only relies on an adversarial objective. We also observe a visually more faithful orientations estimation by our method in the splenium of the corpus callosum and in the pons. In Figure 10, we compare the real GFA map of a test subject to the generated maps by the tested methods. We also compare the real and synthesized GFA maps to the associated HR T1w image of this said subject. It can be seen from Figure 10 that our method generates GFA maps that are more consistent with the real HR T1w image of the subject while visually reducing boundary artifacts. Our method also seems to recover fine anatomical details that are present in the HR T1w but not in the real diffusion. Indeed, the GFA maps generated by our method exhibit sharper edges and better tissue delineation particularly at the bottom of the coronal slice where the real diffusion may lack details. In Figure 11, we compare the ODFs produced by our method and baselines in a region with crossing fibers. One can see that our method generates ODFs that smoothly transition between orientations and plausible crossing estimation. While all the compared methods are able to recover crossing fibers patterns, MA-WGAN seems to generates more of them. However, most of the generated crossing fibers by MA-WGAN can't be found in the real diffusion and their orientation are most of the time far from the ground-truth orientation as shown by the low cosine similarity reported in Table 1.

### 3.8. Streamlines length and volume

**Figure 12:**
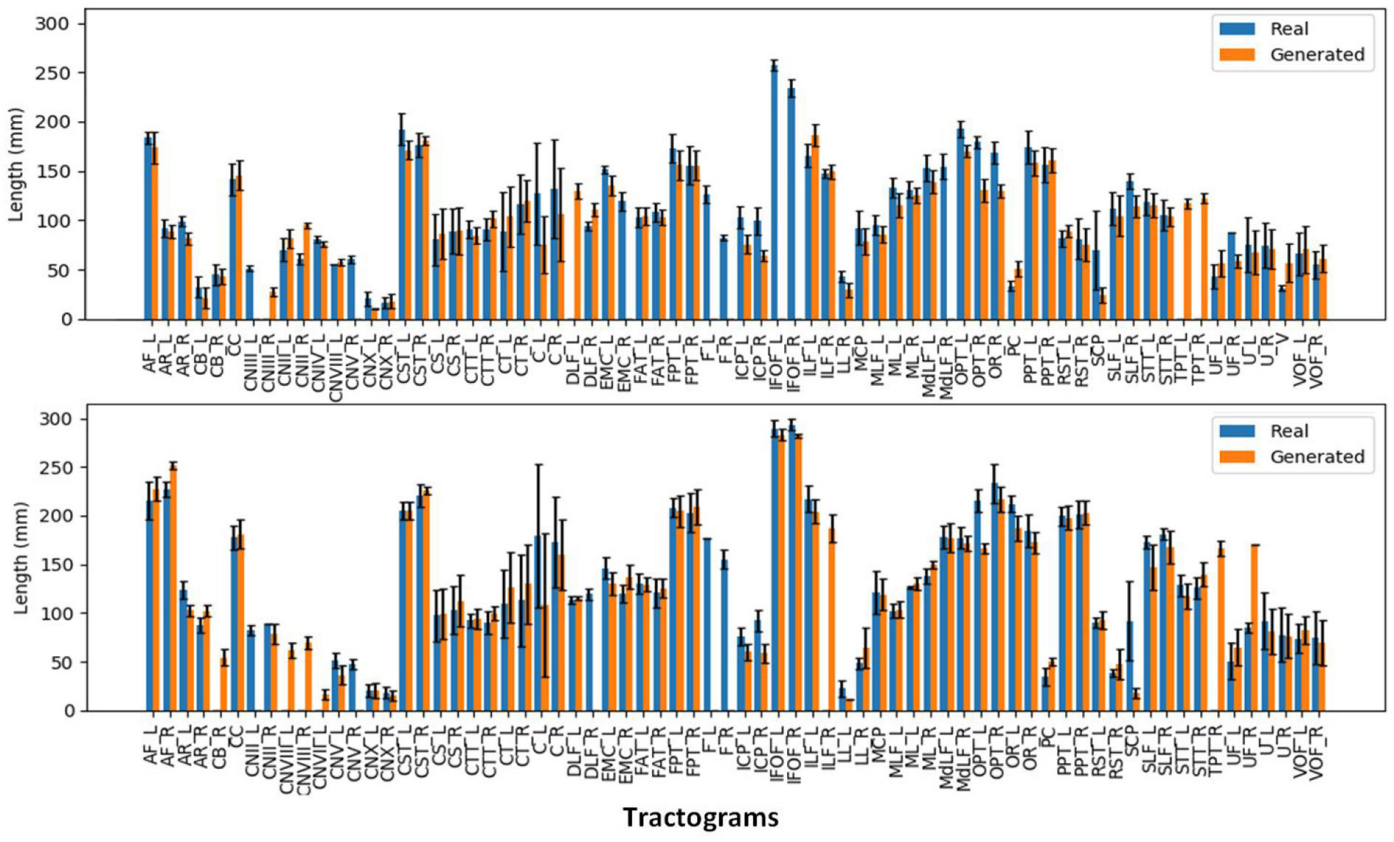
Mean streamline length (in mm) for the segmented bundles from DT tractography (top) and ODF tractography (bottom).

We can observe from the results that reconstructed whole brain tractograms are very similar in size, but that individual bundles segmented from synthesized data tend to be shorter. Indeed, looking at the mean bundle lengths and volumes reported in Table 2, it can be seen that bundles segmented from the generated DT/ODF tractograms are, respectively 14.98 and 2.32% shorter than real data. Nonetheless, generated ODFs tend to produce bundles that are slightly more voluminous with mean volume of ~24,408 voxels compared to ~24,210. In addition, we detail in Figure 12 the mean streamline length (in mm) for each segmented bundles. While some bundles were only segmented on the real data or the generated data (IFOF vs. TPT, for example), we can observe that the bundles recovered in both cases exhibit similar statistics.

**Table 2:** Measures on the real and generated segmented bundles from tractography.

		**Real** **(mean ± std)**	**Generated** **(mean ± std)**
DT	Length (mm)	99.99 ± 54.37	85.01 ± 50.02
	Volume (voxels)	15963.62 ± 24199.55	14119.52 ± 24274.11
ODF	Length (mm)	120.94 ± 72.29	118.13 ± 71.57
	Volume (voxels)	24210.36 ± 42082.48	24408.78 ± 44255.32

### 3.9. Streamlines shape

Despite their similarity in length and volume, the space occupied by the segmented bundles from real and generated data may vary. This disparity can be observed in Figure 7 where we compare three real and generated segmented bundles. Furthermore, we observe a high inter-bundle metrics performance variability. For instance, bundles such as the Frontal Aslant Tract (FAT), Frontopontine (FPT), and Vertical Occipital Fasciculus (VOF) reach a high agreement (i.e., Dice, OL and OR close to 1) whereas the Superior Cerebellar Peduncle (SCP) and Occipitopontine Tract (OPT) are hardly matched. This high inter-bundle variability can be appreciated in Table 3 where we note the mean Dice, OL and OR and their standard deviation for all segmented bundles.

**Table 3:** Average metrics from the bundle shape comparison between real and synthesized data.

	**Dice** **(mean ± std)**	**OL** **(mean ± std)**	**OR** **(mean ± std)**
DT	0.46 ± 0.24	0.38 ± 0.21	1.36 ± 0.86
ODF	0.54 ± 0.24	0.42 ± 0.19	1.23 ± 0.58

From Table 3, we can see that the segmented bundles from the generated ODF are more faithful to their real counterpart than DT with a mean Dice of 0.54 ± 0.24, a mean OL of 0.42 ± 0.19 and a mean OR of 1.23 ± 0.58.

More figures comparing the volume (in voxels) and the shape of each segmented bundles from real and generated diffusion can be found in the Supplementary materials of the manuscript.

## 4. Discussion

In this work, we propose a novel Riemannian deep learning architecture for the synthesis of 3D manifold-valued data and have tested its performance on two tasks: (1) the generation of diffusion tensors (DTs) and (2) the generation of diffusion orientation diffusion functions (ODFs). Specifically, we have explored the feasibility of generating high-resolution DT and ODF from high-resolution structural T1w images and unpaired LR diffusion. We demonstrate that a standard model relying on Euclidean operations fails to capture the geometry of the diffusion data manifold which leads to the estimation of physically incorrect diffusion (i.e., 3,843.5 ± 481.22 non-SPD tensors and 1,559.7 ± 122.3 non-PDF ODF on average). To alleviate this issue, we have built a framework on top of recent advances in manifold-valued data processing and Riemannian geometry (Arsigny et al., 2006; Cheng et al., 2009; Huang et al., 2019) to ensure the validity of the generated diffusion. We have evaluated the generated volumes properties using mean squared errors of FA/GFA maps, geodesic distances and cosine similarities between real and predicted principal fiber orientation. To further evaluate the integrity of the synthesized diffusion in a typical diffusion application, we have performed tractography and assessed the lengths, volumes and shapes of resulting tractrograms and segmented bundles.

### 4.1. Structural-to-diffusion synthesis performance

The generation of DT/ODF solely relying on a T1w image is an ill-posed problem for which a single T1w intensity can correspond to several fiber arrangements. However, by providing the contextual information required for the network to localize the structural input, we observe that strong fiber patterns can successfully be recovered by our method and baselines. Hence, it is reasonable to say that there exists a relationship between the high-level geometry of the brain and its underlying fibers organization. This seems to be particularly true in regions of higher anisotropy where fiber tracts are strongly organized. As a result, we observed a better estimation of the principal fibers orientation in regions with higher FA/GFA and a generally poor estimation of the principal orientations in regions with a more chaotic distribution of orientations like in the ventricles.

Using HR structural images to drive the synthesize of diffusion can help recovering some fine anatomical details and sharp edges. By leveraging the detailed information contained in HR structural images, our network is not limited by the coarseness of the low-resolution input diffusion signal such as in interpolation-based methods. This transfer of information from HR to LR images is enforced by our cycle-consistency loss that preserves a high structural coherence between the HR structural inputs and the generated diffusion. In addition, our adversarial and prior loss help recovering plausible fiber patterns better than our baselines by leveraging unpaired examples of real diffusion.

Furthermore, the manifold-awareness yields on-par or better metrics when compared with equivalent Euclidean architectures. The manifold-awareness, however, comes with the benefit of ensuring the mathematical properties of the synthesized diffusion regardless of the amount of network training.

### 4.2. Tractography performance

#### 4.2.1. DT vs. ODF

We observe from Table 2 that bundles segmented from ODF tractography tend to be longer and more voluminous which is usually an expected behavior. While a thorough comparison between DT and ODF tractography is out-of-scope for this work (c.f. Farquharson et al., 2013; Thomas et al., 2014; Jeurissen et al., 2019), we can nevertheless appreciate that the synthesized DT and ODF behave in a manner similar to their real counterparts in the context of tractography.

Moreover, we observed that the bundles segmented from ODF tractography have a higher mean Dice, higher overlap and lower overreach than bundles segmented from DT tractography. Since the same algorithm was used to perform tractography on both datasets, the main difference is that DT tractography makes use of a single direction while ODF tractography may use multiple local maxima to propagate streamlines. As such, the lower discrepancies in ODF bundle shapes can be explained by the multiple directions used in tractography compensating for local errors. At the opposite, DT tractography is known to be sensitive to local estimation error (Huang et al., 2004). This sensitivity, which often lead to the early termination or to the switch to a wrong adjacent tract of the tracking algorithm (Jeurissen et al., 2019), can greatly affect the final tractograms shape.

#### 4.2.2. Bundle shape analysis

Since DT/ODF synthesis studies are still few, it is hard to provide a definitive conclusion on the quality of the streamlines generated on synthesized data. However, we observe that whole brain tractograms have a similar streamline length, occupy the same number of voxels and have the same shape. Segmented bundles, if extracted from both real and generated data, also tend to exhibit the same average length and volume.

While the reported bundle shape metrics might seem to indicate a poor correspondence between bundles generated from real and synthesized data, it should be noted that bundle segmentation is a highly variable operation. For example, Rheault et al. (2020), which analyzed the reproducibility of the segmentation of a single bundle reports a median Dice scores around 0.77 for intra-rater reproducibility, 0.65 for inter-rater reproducibility and 0.8 for reproducibility with a gold standard. In a similar manner, Schilling et al. (2021) analyzed the variability in the segmentation of 14 bundles between 42 groups using both manual and automatic segmentation. While few actual metrics are reported, figures indicate a generally low Dice score, as well as a high variability in bundle volume and streamline length for inter-protocol responsibility. Analysis for specific pathways report Dice scores between 0.4 and 0.6 for inter-protocol and inter-subject reproducibility and Dice scores between 0.6 and 0.8 for intra-protocol reproducibility. As such, we can theorize that the reported Dice scores in the present work could be impacted by the inherent variability in the segmentation process.

## 5. Limitations

Our work has shown that the optimization of log-Euclidean metrics in diffusion tensors and ODFs indeed reduces the diffusion synthesis error within a local region, typically within a patch, but does not take into account global information at higher levels such as the global brain connectivity. Our approach, therefore, enables local approximation of the diffusion to be more accurate, however, it still lacks support of a global optimization at the whole connectome level. Long fiber tracts spanning multiple patches, such as IFOF, remains harder to synthesize accurately. Synthesis has also been only tested on healthy subjects, for the purpose of detecting deviations from healthy fiber tracts. The generalization of the method on synthesizing diffusion imaging from structural images under neurological conditions, such as a brain tumor, is left as future work.

## 6. Conclusion

This work presents a novel Riemannian network architecture for the cycle-consistent synthesis of diffusion tensors and diffusion ODF in high-resolution structural T1w space. The results have demonstrated that our Riemannian architecture can synthesize mathematically valid diffusion images with a 5% improvement in principal fibers orientation and a 23% improvement in FA MSE with respect to our baselines. The better performance of our approach over compared methods shows the benefit of using both paired and unpaired samples in a single objective. Furthermore, as opposed to standard Euclidean deep learning models, which generate an average of 3,844 invalid tensors and 1,560 invalid ODFs per volume, our method guarantees the mathematical coherence of the synthesized diffusion schemes, free of invalid tensors or ODFs.

Moreover, we have evaluated qualitatively our generated diffusion volumes by comparing their tractograms with their real counterparts. It was observed that our generated T1w-driven diffusion shares similarities with the real diffusion in terms of streamline length, volume and fiber bundles shape. We have also shown the ability of our network to transfer fine anatomical details from the high-resolution T1w images to diffusion images. This transfer of information allows the generation of images with sharper edges and a higher level of details that could not be achieved with image interpolation.

Our results suggest that the high-level geometry of the brain, encoded in structural T1w images, can be used to predict its global fiber bundles organization. Leveraging this principle, our method could enable the fast synthesis of DT and ODF in situations where the acquisition of diffusion imaging is not available. More generally, it offers the basis of a framework targeting any real-to-manifold-valued image translation tasks. For instance, our method could be used for missing modalities synthesis and datasets completion, manifold-valued image inpainting or manifold-valued population based statistics that rely on non-Euclidean metrics.

## Data availability statement

Publicly available datasets were analyzed in this study. This data can be found at: Human Connectome Project (HCP), https://www.humanconnectome.org/.

## Ethics statement

Ethical review and approval was not required for the study on human participants in accordance with the local legislation and institutional requirements. Written informed consent from the patients/participants or patients/participants' legal guardian/next of kin was not required to participate in this study in accordance with the national legislation and the institutional requirements.

## Author contributions

BA-R, CD, and HL conceived the method. BA-R implemented all visualizations, the experimental procedures, and wrote the first draft of the manuscript. AT performed the tractography, the tractography analysis, and contributed to the tractography related parts of the manuscript. AT, CD, HL, P-MJ, and MD reviewed and edited the manuscript. This work was supervised by HL and CD. All authors contributed to the article and approved the submitted version.

## Funding

This work was supported financially by the Canada Research Chair on Shape Analysis in Medical Imaging, the Research Council of Canada (NSERC), the Fonds de Recherche du Québec (FQRNT), the Réseau de Bio-Imagerie du Québec (RBIQ), and ETS Montreal.

## Conflict of interest

The authors declare that the research was conducted in the absence of any commercial or financial relationships that could be construed as a potential conflict of interest.

## Publisher's note

All claims expressed in this article are solely those of the authors and do not necessarily represent those of their affiliated organizations, or those of the publisher, the editors and the reviewers. Any product that may be evaluated in this article, or claim that may be made by its manufacturer, is not guaranteed or endorsed by the publisher.
