# Supplementary material for: Manifold-aware synthesis of high-resolution diffusion from structural imaging
Source: Front Neuroimaging. 2022 Sep 8;1:930496. doi: 10.3389/fnimg.2022.930496 (PMC10406190; doi:10.3389/fnimg.2022.930496)
Supplement: Supplementary file 1 [file Data_Sheet_1.PDF]

frontiers

Supplementary Material

1 SUPPLEMENTARY TABLES

Table S1. The cosine similarity between the principal orientation obtained by our method with different amount paired subjects used as prior. Since the metrics are more relevant in regions that typically encode fibers information, we report them at increasing FA thresholds of 0.2 and 0.5.

Paired Subjects	Cosine Sim (DT)		Cosine Sim (ODF)	
	FA ≥ 0.2	FA ≥ 0.5	GFA ≥ 0.2	GFA ≥ 0.5
0	0.7315	0.7955	0.7029	0.7380
10	0.7795	0.8599	0.8664	0.9058
25	0.8152	0.8863	0.8745	0.9081
**50**	**0.8648**	**0.9167**	**0.8846**	**0.9425**
75	0.8317	0.8993	0.8674	0.9051

Table S2. The amount of non-SPD tensors and non-PDF dODFs generated by the compared methods for five randomly selected test subjects. The manifold-awareness ensures that the generated diffusion schemes lie on their respective data manifold.

Method	Manifold-Awareness	Non-SPD Tensors	Non-PDF ODF
U-Net	✗	3843.5 ± 481.22	1559.7 ± 122.3
**MA-U-Net**	✓	**0**	**0**
**MA-WGAN**	✓	**0**	**0**
**Ours**	✓	**0**	**0**

2 BACKPROPAGATION FOR DIFFUSION TENSORS LEARNING

The $\log_{\mathrm{Id}}(\,\cdot\,)$ and $\exp_{\mathrm{Id}}(\,\cdot\,)$ maps ensure that our generator network synthesizes valid diffusion tensors. Both maps involves the EIG operator on 3×3 symmetric matrices whose gradients must be defined to train the network with the standard backpropagation algorithm. Employing the matrix generalization of backpropagation (Ionescu et al., 2015; Huang and Van Gool, 2017), we define the partial derivatives of the objective function at the kth layer with respect to a generated diffusion tensor $\mathbf{M}$ as follows:

$$\forall \mathbf{M}_{k-1} \in \mathbf{X_{k-1}}, \frac{\partial L^{(k)}}{\partial \mathbf{M}_{k-1}} = 2\mathbf{U}\left(\mathbf{K}^\top \circ \left(\mathbf{U}^\top \frac{\partial L^{(k^{'})}}{\partial \mathbf{U}}\right)_{sym}\right)\mathbf{U}^\top\right) + \mathbf{U}\left(\frac{\partial L^{(k^{'})}}{\partial \mathbf{\Sigma}}\right)_{diag}\mathbf{U}^\top, \tag{S1}$$

with

$$\mathbf{M}_{k-1} = \mathbf{U}\mathbf{\Sigma}\mathbf{U}^\top, \tag{S2}$$

and

$$\mathbf{K}(i,j) = \begin{cases} \frac{1}{\sigma_i - \sigma_j} & i \neq j \\ 0 & i = j, \end{cases} \tag{S3}$$

where $\mathbf{A}_{sym} = \frac{1}{2}(\mathbf{A} + \mathbf{A}^\top)$ and $\mathbf{A}_{diag}$ is $\mathbf{A}$ with nonzero elements only in its diagonal. Furthermore, $\mathbf{X_{k-1}} \in \mathbb{R}^{B \times 9 \times D \times H \times W}$ is a batch of 3D DT patches with size $D \times H \times W$ and $\mathbf{K}$ is built upon the eigenvalues σ in $\mathbf{\Sigma}$ of $\mathbf{M}$.

The variations $\frac{\partial L^{(k^{'})}}{\partial \mathbf{U}}$ and $\frac{\partial L^{(k^{'})}}{\partial \mathbf{\Sigma}}$ in Eq. (S1) are defined for every tensor $\mathbf{M}$ for both maps $\log_{\mathrm{Id}}(\cdot)$ and $\exp_{\mathrm{Id}}(\cdot)$ respectively as:

$$\frac{\partial L^{(k^{'})}}{\partial \mathbf{U}} = 2\left(\frac{\partial L^{(k+1)}}{\partial \mathbf{M_k}}\right)_{sym} \mathbf{U}\log(\mathbf{\Sigma}), \tag{S4}$$

$$\frac{\partial L^{(k^{'})}}{\partial \mathbf{\Sigma}} = \mathbf{\Sigma}^{-1}\mathbf{U}^\top\left(\frac{\partial L^{(k+1)}}{\partial \mathbf{M_k}}\right)_{sym} \mathbf{U}, \tag{S5}$$

and

$$\frac{\partial L^{(k^{'})}}{\partial \mathbf{U}} = 2\left(\frac{\partial L^{(k+1)}}{\partial \mathbf{M_k}}\right)_{sym} \mathbf{U}\exp(\mathbf{\Sigma}), \tag{S6}$$

$$\frac{\partial L^{(k^{'})}}{\partial \mathbf{\Sigma}} = \exp(\mathbf{\Sigma})\mathbf{U}^\top\left(\frac{\partial L^{(k+1)}}{\partial \mathbf{M_k}}\right)_{sym} \mathbf{U}. \tag{S7}$$

With the help of Eq. (S1) and the successive application of the $\exp_{\mathrm{Id}}(\cdot)$ and $\log_{\mathrm{Id}}(\cdot)$ maps, we make sure that the generated tensors are and stay SPD throughout the training. An open-source implementation of these differentiable functions is available on our Github [1,2].

3 TRACTOGRAPHY ASSESSMENT

This appendix presents additional figures that compare the generated and the expected real tractograms. First, we compare in Figure S1 the mean volume, streamline length, Dice, OL and OR when considering the whole brain tractograms.

We then compare the mean volume of each recovered bundle in Figure S2 below:

[1] https://github.com/banctilrobitaille/torch-vectorized

[2] https://torch-vectorized.readthedocs.io/en/latest/

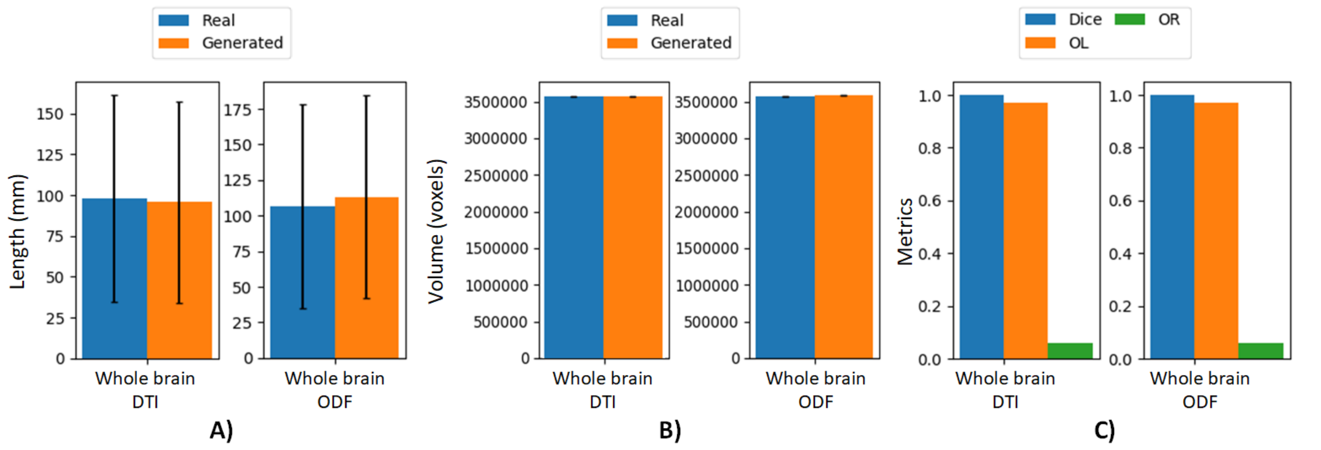

Figure S1. In **A)**, the mean streamline length (in mm). In **B)**, the mean volume (in voxels) and in **C)**, the mean Dice, OL and OR.

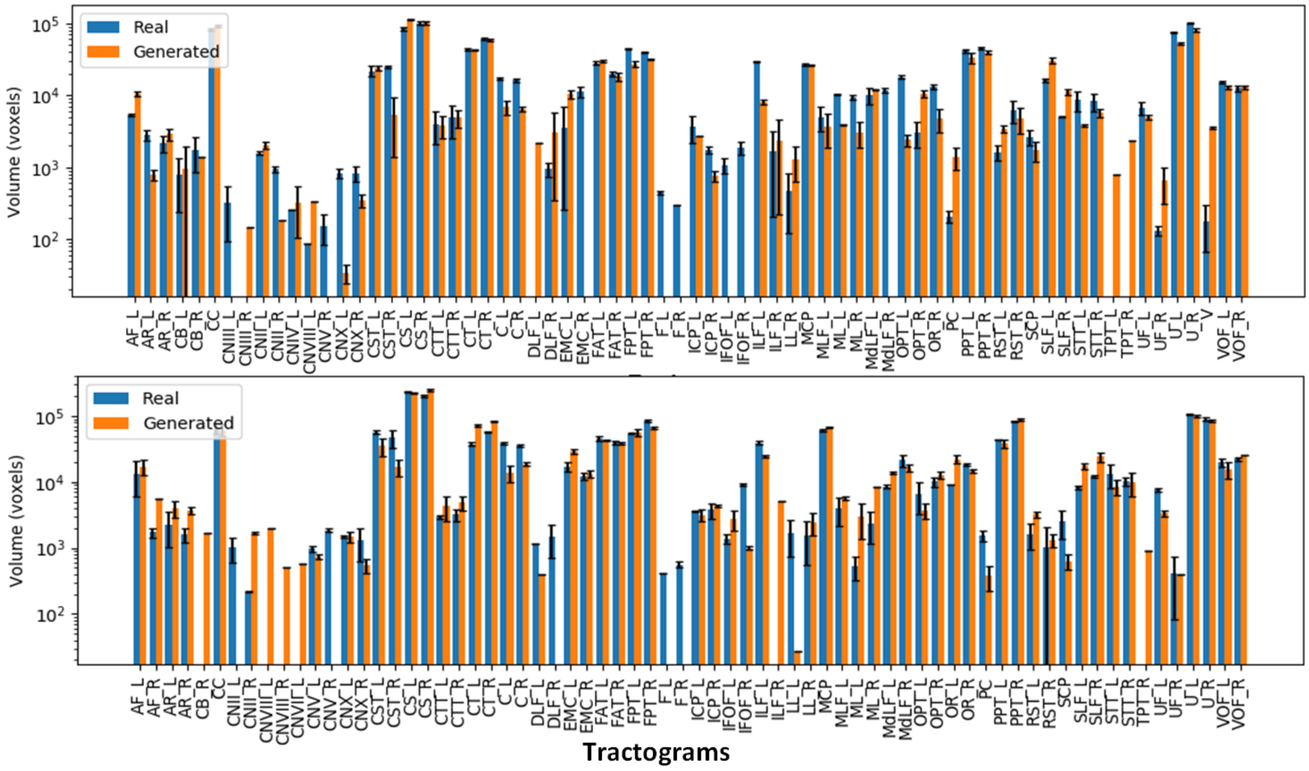

Figure S2. **Top row**: Mean bundle volumes (in voxels) from real and generated DT. **Bottom row**: Mean bundle volumes (in voxels) from real and generated ODF.

Finally, Figure S3 presents the Dice, OL and OR yielded by each recovered bundles.

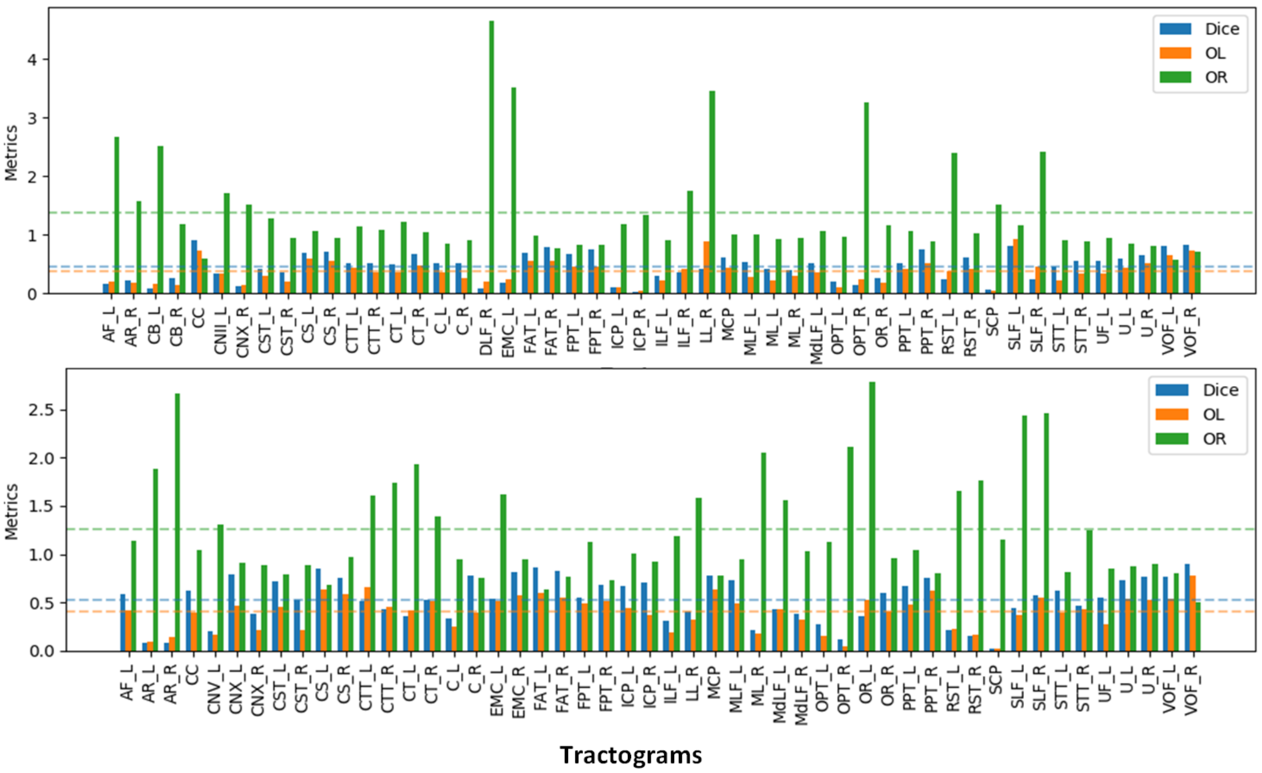

Figure S3. Top row: Dice, OL and OR between bundles and tractograms from real and generated DT. **Bottom row**: Dice, OL and OR between bundles and tractograms from real and generated ODF. The lines indicate the mean metric values for all bundles.

4 VISUAL ASSESSMENT OF SYNTHESIZED VOLUMES

We provide, in the following appendix, the FA and color FA maps of five additional test subjects. We first compare in Figure S4 the generated and real FA of a sagittal, axial and coronal slice. We then compare in Figure S5 the generated and real color FA of the same five subjects. One can appreciate how our method adapts to different brain geometries and generates plausible FA and principal orientations despite the differences in the anatomy of the subjects.

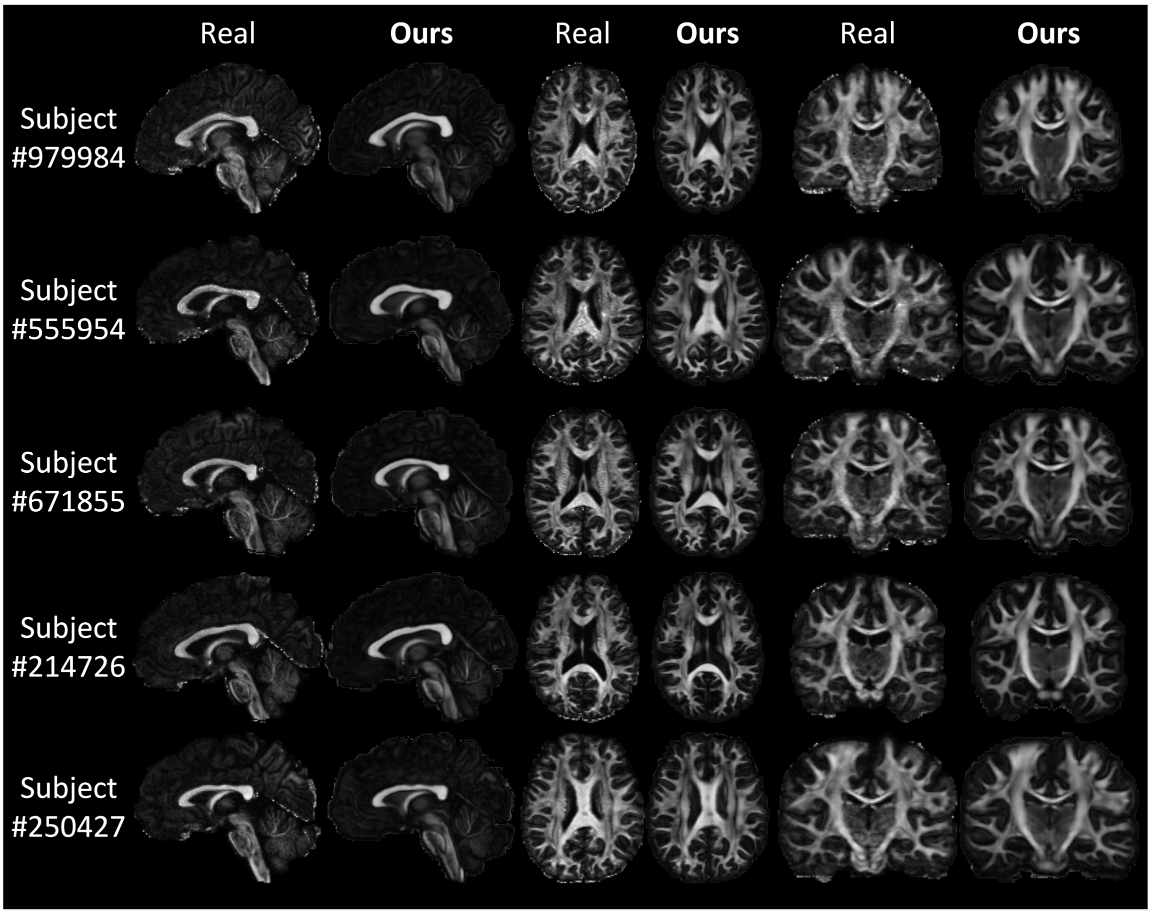

Figure S4. The real and synthesized FA maps of five test subjects in the sagittal, axial and coronal planes.

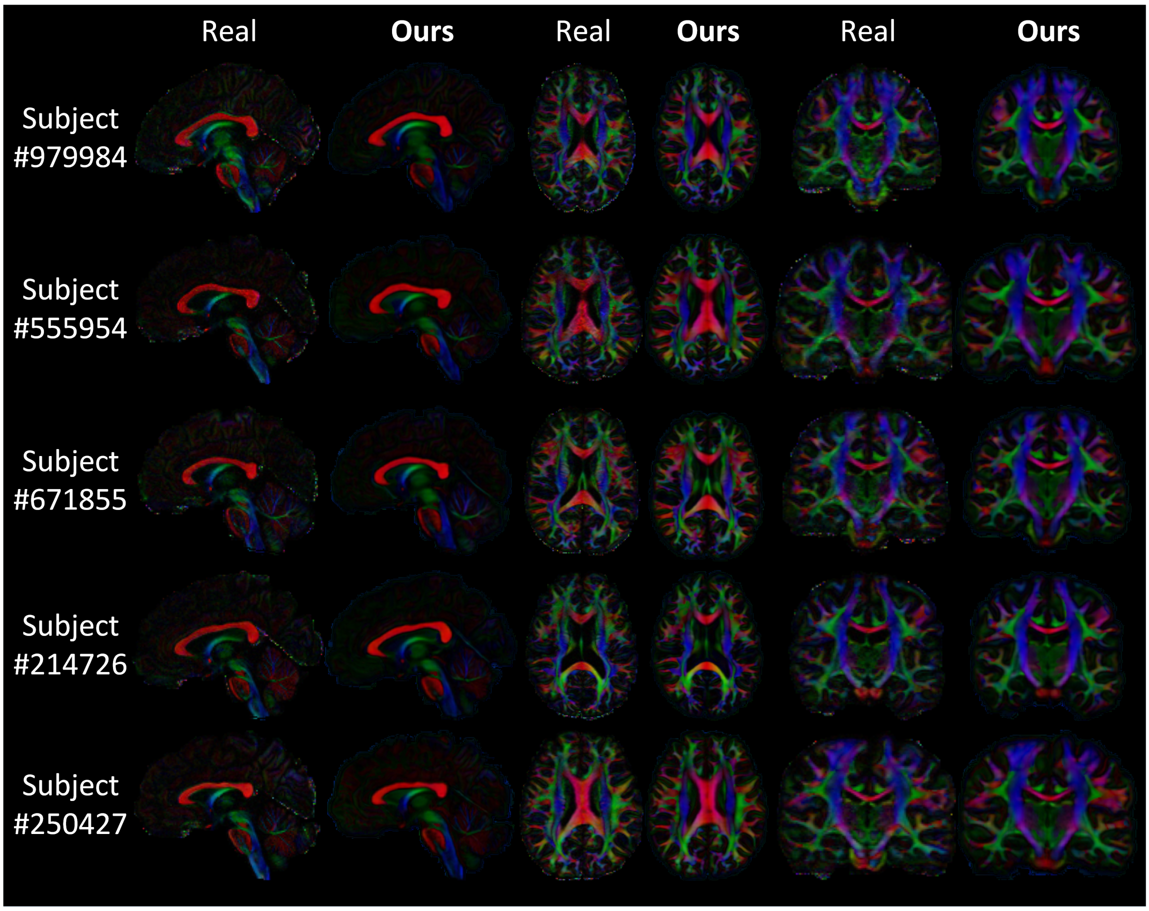

Figure S5. The real and synthesized color FA maps of five test subjects in the sagittal, axial and coronal planes.

REFERENCES

Ionescu C, Vantzos O, Sminchisescu C. Matrix backpropagation for deep networks with structured layers. *Proceedings of the IEEE International Conference on Computer Vision (ICCV)* (2015), 2965–2973.

Huang Z, Van Gool L. A riemannian network for SPD matrix learning. *31st AAAI Conference on Artificial Intelligence, AAAI 2017* (2017).
